# Total Syntheses and Preliminary Biological Evaluation of Brominated Fascaplysin and Reticulatine Alkaloids and Their Analogues

**DOI:** 10.3390/md17090496

**Published:** 2019-08-25

**Authors:** Maxim E. Zhidkov, Polina A. Smirnova, Oleg A. Tryapkin, Alexey V. Kantemirov, Yuliya V. Khudyakova, Olesya S. Malyarenko, Svetlana P. Ermakova, Valeria P. Grigorchuk, Moritz Kaune, Gunhild von Amsberg, Sergey A. Dyshlovoy

**Affiliations:** 1 Department of Organic Chemistry and Laboratory of Biologically Active Compounds, School of Natural Sciences, Far Eastern Federal University, 8 Sukhanov Str., Vladivostok 690950, Russia; 2 G.B. Elyakov Pacific Institute of Bioorganic Chemistry, 159 Prospekt 100 Let Vladivostoku, Vladivostok 690022, Russia; 3 Federal Scientific Center of the East Asia Terrestrial Biodiversity (Institute of Biology and Soil Science), Far Eastern Branch of the Russian Academy of Sciences, 159 Prospect 100-Let Vladivostoku, Vladivostok 690022, Russia; 4 Department of Oncology, Hematology and Bone Marrow Transplantation with Section Pneumology, Hubertus Wald-Tumorzentrum, University Medical Center Hamburg-Eppendorf, 20246 Hamburg, Germany; 5 Martini-Klinik Prostate Cancer Center, University Hospital Hamburg-Eppendorf, 20246 Hamburg, Germany

**Keywords:** total synthesis, 14-bromoreticulatine, 3,10-dibromofascaplysin, bioactivity

## Abstract

A simple approach toward the synthesis of the marine sponge derived pigment fascaplysin was used to obtain the marine alkaloids 3-bromofascaplysin and 3,10-dibromofascaplysin. These compounds were used for first syntheses of the alkaloids 14-bromoreticulatate and 14-bromoreticulatine. Preliminary bioassays showed that 14-bromoreticulatine has a selective antibiotic (to *Pseudomonas aeruginosa*) activity and reveals cytotoxicity toward human melanoma, colon, and prostate cancer cells. 3,10-Dibromofascaplysin was able to target metabolic activity of the prostate cancer cells, without disrupting cell membrane’s integrity and had a wide therapeutic window amongst the fascaplysin alkaloids.

## 1. Introduction

**Figure 1:**
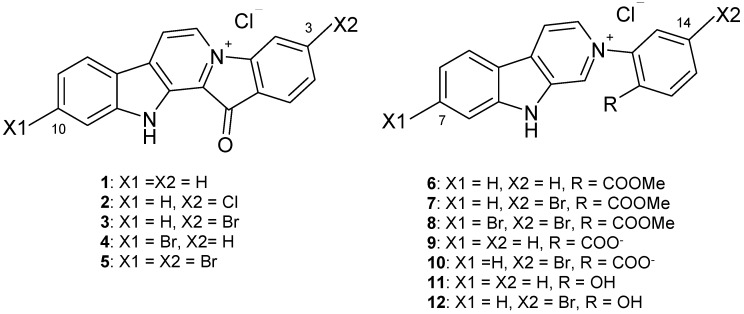
Structures of fascaplysin (**1**) and its derivatives (**2**–**5**); reticulatine (**6**), 14-bromoreticulatine (**7**), 7,14-dibromoreticulatine (**8**), reticulatate (**9**), 14-bromoreticulatate (**10**), reticulatol (**11**), 14–bromoreticulatol (**12**).

Fascaplysin, homofascaplysins A–C, and their brominated analogues form the group of marine alkaloids based on the 12*H*-pyrido [1–2-*a*:3,4-*b′*] diindole ring system [1]. The red pigment fascaplysin (**1**, Figure 1) is the first isolated compound among these alkaloids and at the present time is the most investigated one [2]. This compound could be used in the field of medicinal chemistry due to a broad range of bioactivities including antibacterial, antifungal, antiviral, and antimalarial properties. In addition, it is able to inhibit the proliferation of numerous cancer cell lines and reveals anti-angiogenesis properties on human umbilical vein endothelial cells (HUVEC) [3,4,5,6,7,8,9]. Fascaplysin suppresses the growth of S180 cell-implanted tumors in vivo [10]. Remarkably, it effectively decreases the growth of small cell lung cancer (SCLC) spheroids derived from circulating tumor cells. In fact, high numbers of circulating tumor cells are linked to the dismal prognosis of SCLC [11]. Its mechanisms of action include the selective inhibition of cyclin-dependent kinase 4, which regulates the G0–G1/S checkpoint of the cell cycle, the intercalation of DNA, and the induction of apoptosis, partially, as a result of the activation of the TRAIL signaling pathway by the upregulation of DR5 expression [12,13,14,15]. It was also found that fascaplysin induced autophagy as a cytoprotective response via ROS and p8 in vascular endothelial cells (VECs) [16]. A cooperative interaction between apoptotic and autophagic pathways is exhibited by fascaplysin through the inhibition of PI3K/AKT/mTOR signaling cascade in human leukemia HL-60 cells [17]. It also causes the downregulation of survivin and HIF-1α and inhibition of VEGFR2 and TRKA, and sensitizes anti-cancer effects of drugs targeting AKT and AMPK [18,19]. Fascaplysin could be used as a P-gp inducer for the development of anti-Alzheimer agents [20]. It may also serve as a “balanced agonist” of the µ-opioid receptor with a signaling profile that resembles endorphins [21].

Remarkably, some derivatives of fascaplysin were found to have an increased therapeutic potential compared to the parental alkaloid. Thus, methylation of fascaplysin at C-9 results in the more potent Aβ aggregation inhibitor than alkaloid **1** [22]. The synthetic chloro derivative of fascaplysin (**2**) inhibited the VEGF-mediated microvessel sprouting with blood vessel formation in the matrigel plug of C57/BL6J mice and the tumor growth in ET (solid) mouse tumor model [23]. In addition, natural 3- and 10-bromofascaplysins (**3**,**4**) showed anti-cancer activity at submicromolar concentrations. This was, at least in part, mediated through the induction of caspase-8, -9, and -3-dependent apoptosis [24]. Antitumor effects of 3-bromofascaplysin and 10-bromofascaplysin were comprehensively examined in an in vitro glioma C6 cell model. The cytotoxic efficiency of compounds **3** and **4** was higher than that of unsubstituted fascaplysin; 3-bromofascaplysin exhibited the best capacity to kill glioma C6 cells [25]. 3,10-Dibromofascaplysin (**5**)—the last representative of fascaplysin alkaloids was synthesized in eight steps from 6-bromoindole and 4-amino-2-bromotoluene, but the therapeutic potential of that perspective compound has not been investigated yet [26].

Herein, we report the two-step method for the syntheses of 3-bromofascaplysin and 3,10-dibromofascaplysin, which has been previously used for the synthesis of fascaplysin. The similarity in structures lets us to use these compounds as starting materials for the first syntheses of several alkaloids of reticulatine group (compounds **6**–**12**, Figure 1, [27]). Also, the bioactivities of the obtained compounds were investigated.

## 2. Results

### 2.1. Chemistry

**Scheme 1:**
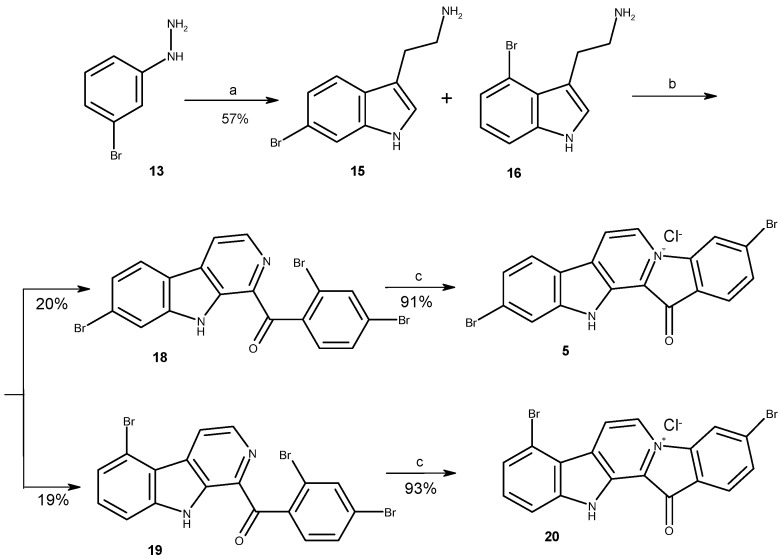
Reagents and conditions. (**a**) 4-bromobutanal (**14**, 4.0 equiv.), EtOH, H_2_O, autoclave, 150 °C, 1 h; (**b**) 2,4-dibromoacetophenone (**17**) (1 equiv.), I_2_ (0.8 equiv.), DMSO, 110 °C, 1 h, then tryptamines **15**, **16** (1.0 equiv.), DMSO, 110 °C, 4 h; (**c**) 220 °C, 15 min, then HCl (aq).

Several groups have synthesized fascaplysin and its naturally occurring analogs and more than 10 syntheses have been reported to date [28,29,30,31,32,33,34,35,36,37,38]. Among them the two-step scheme by Zhu et al. is the most suitable for the preparation of the target compounds [36]. To apply that synthetic scheme for the synthesis of 3,10-dibromofascaplysin, the reaction between 3-bromophenylhydrazine (**13**) and 4-bromobutanal (**14**) in an autoclave at 150 °C was used to prepare the mixture of 6-bromotryptamine (**15**) and 4-bromotryptamine (**16**) (Scheme 1). Thereafter, the obtained mixture and 2,4-dibromoacetophenone (**17**) were subjected to the cascade coupling protocol, previously developed by Zhu et al., which included the sequential iodination of the corresponding acetophenone, the Kornblum oxidation of the intermediate in the presence of DMSO to phenylglyoxal, and its Pictet–Spengler condensation with the derivative of tryptamine followed by the oxidation of the intermediate. After chromatography purification, two isomeric 1-benzoyl-β-carbolines (**18**, **19**) were obtained with the yields of 20% and 19%, respectively. These products were subsequently transformed to 3,10-dibromofascaplysin (**5**) and its isomer **20** according to the procedure reported by the group of Radchenko [31].

**Scheme 2:**
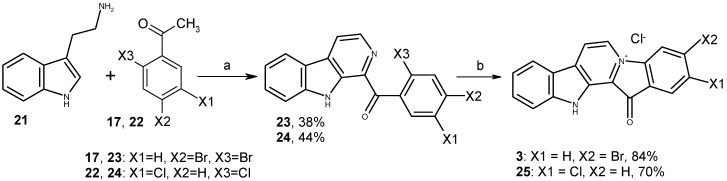
Reagents and conditions. (**a**) I_2_ (0.8 equiv.), DMSO, 110 °C, 1 h, then tryptamine (1.0 equiv.), DMSO, 110 °C, 4 h; (**b**) 220 °C, 15 min, then HCl (aq).

3-Bromofascaplysin was prepared in a similar manner from tryptamine (**21**) and 2,4-dibromoacetophenone (**17**) with a total yield of 32%. Taking into account the high biological activity of synthetic chloro derivatives of fascaplysin, we obtained the corresponding derivative at C-2 (**25**) from tryptamine and 2,5-dichloroacetophenone (**22**) by a similar method (Scheme 2) [20].

**Scheme 3:**
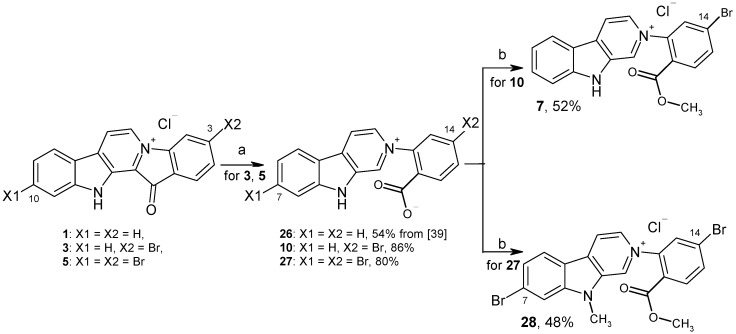
Reagents and conditions–(**a**) NaOH (4 equiv.), DMF, r.t., 0.5 h; (**b**) (CH_3_)_2_SO_4_ (4 equiv.), CH_3_CN, 1 h.

Previously zwitter-ionic β-carboline **26** was obtained from fascaplysin that was treated with aqueous solution of NaOH or 30% NH_4_OH [39]. After optimization of the reaction conditions 14-bromoreticulatate (**10**) and its dibromo analog (**27**, not isolated from marine organisms) were obtained from compounds **3** and **5** in DMF at r.t. with 86% and 80% yields, respectively (Scheme 3). Different conditions for methylation of compounds **10** and **27** were investigated, including (i) the interaction with diazomethane; (ii) with POCl_3_ and following treatment with methanol; (iii) the reaction with dimethyl sulfate. In the latter case, best results were achieved. However, 7,14-dibromoreticulatine (**8**) was not obtained after methylation of compound **27**. Instead, the product of dimethylation (**28**) was obtained. Because of the insolubility of compound **28** in most solvents, only MS and ^1^H NMR were used to identify its structure. The spectral characteristics of synthetic 3-bromofascaplysin, 3,10-dibromofascaplysin, 14-bromoreticulatate, and 14-bromoreticulatine were identical to those of the natural products.

### 2.2. Biology

The bioactivities of obtained compounds were investigated using fascaplysin (**1**) as a standard. First, the cytotoxic effects of the compounds against human colorectal carcinoma (HT-29), human breast cancer (T-47D), and melanoma (SK-MEL-28) cell lines were evaluated by MTS assay (Table 1). The cells were incubated with different concentrations of the respective compounds (0–5 µM) for 24 h. The concentration that caused inhibition of 50% of cell viability (IC_50_) was 5 µM for compound **1** against T-47D cells. Other investigated compounds were less cytotoxic against this type of cancer cells at concentrations up to 5 µM. However, the IC_50_ of **1**, **3**, and **7** were detected at concentrations ranging from 1.1 to 1.9 µM against SK-MEL-28 cells. Among the investigated cancer cells, the most resistant cell line to the cytotoxic effect of the compounds was found to be breast cancer cells T-47D, while the most sensitive were melanoma cells SK-MEL-28. It was shown that compounds **1** and **3** possessed comparable IC_50_ against colorectal carcinoma cells HT-29. Our results indicated that the investigated compounds reveal selective cytotoxic effects to different cancer cell lines, with highest efficacy in melanoma cells SK-MEL-28.

**Figure 2:**
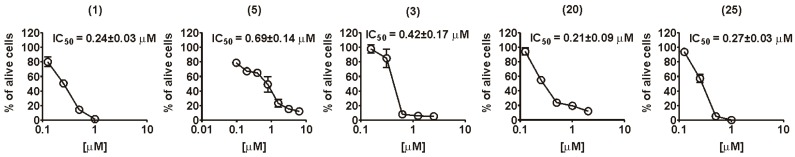
Effect of the compounds on viability of 22Rv1 cells. The effect was accessed using MTT assay. Cells were treated with the compounds for 48 h. The values are presented as mean expression levels ± SD are shown.

We have also investigated the effect of the synthesized compounds on the viability and the growth of human prostate cancer drug-resistant PC-3 and 22Rv1 cells. IC_50_s of the substances have been determined by both, MTT and trypan blue exclusion assay (ViCell assay) (Table 1, Figure 2). It is known that MTT assay accesses the metabolic activity of the cells, while the trypan blue exclusion assay shows the alive cells with either intact (non-stained) or disrupted (stained) membranes. Compound **20** was identified to be the most active among the tested fascaplysin derivatives. However, its cytotoxicity determined by MTT assay was within the range of compounds **3** and **25** and fascaplysin (**1**). Interestingly, compound **5**, having a higher IC_50_ of 0.69 ± 0.14 µM, had a very smooth cytotoxicity profile, suggesting a wide therapeutic window (Figure 2). Moreover, for compound **5**, the IC_50_ determined by trypan blue exclusion assay was ~8-fold higher than the IC_50_ accessed using MTT test. In contrast, for the other compounds the difference of the IC_50_s generated by the two different methods was distinctly less pronounced. This may indicate an antimetabolic effect of compound **5** rather an effect on the cell membrane integrity (necrotic-like cell death). Compound **5** starts to suppress cancer cell viability/proliferation already at 0.1 µM, while the ranges of active concentrations for the other two tested compounds were rather narrow. Fascaplysin (**1**) started to suppress cancer cell viability/proliferation at 0.125 µM. Remarkably, for this compound no difference between IC_50_s generated with the two different methods was observed. The high potential of compound **5** for therapeutic assays was also confirmed by its low cytotoxity (IC_50_ 50 µM) against normal MRC-9 lung cells.

It is known that fascaplysins exhibit potent but nonselective antibiotic activities. To evaluate activity of reticulatines in comparison to known fascaplysin derivatives, compounds **1**, **3**, **7**, **25** were studied in vitro for antibiotic activity against several microbes using the disk diffusion soft agar assay as shown in Table 2. 14-Bromoreticulatine (**7**) showed potent activity against *Pseudomonas aeruginosa* while it exhibited low activity or no activity at all against other tested microbes. As expected, high and non-selective antibiotic activities were demonstrated for the other tested compounds (**1**, **3**, **25**).

## 3. Materials and Methods

### 3.1. Chemistry

All starting materials are commercially available. Commercial reagents were used without any purification. The products were isolated by MPLC: Buchi B-688 pump, glass column C-690 (15 × 460 mm) with Silica gel (particle size 0.015–0.040 mm), UV-detector Knauer K-2001. The analytical examples were purified by Shimadzu HPLC system (model: LC-20AP) equipped with a RID detector (model: RID 10A) using Supelco C18 (5 µm, 4.6 × 250 mm) column using ACN:water (20:80, 50:50, 70:30) mobile phase by isocratic elution at flow rate of 1 mL/min. NMR spectra were recorded with a NMR instrument operating at 400 MHz (^1^H) and 100 MHz (^13^C). Proton spectra were referenced to TMS as internal standard, in some cases, to the residual signal of used solvents. Carbon chemical shifts were determined relative to the ^13^C signal of TMS or used solvents. Chemical shifts are given on the δ scale (ppm). Coupling constants (J) are given in Hz. Multiplicities are indicated as follows: s (singlet), d (doublet), t (triplet), q (quartet), m (multiplet), or br (broadened). The original spectra of the relative compounds could be found in Supplementary Materials. High-resolution mass spectra (HRMS) were obtained with a time-of-flight (TOF) mass spectrometer equipped with an electrospray source at atmospheric pressure ionization (ESI).

#### 3.1.1. Preparation of Mixture of Tryptamines **15** and **16**

A mixture of 4-bromobutanal (1.33 g, 8.8 mmol), 3–bromophenylhydrazine hydrochloride (0.50 g, 2.2 mmol), EtOH (3 mL), and H_2_O (1 mL) was placed into an autoclave and heated at 150 °C for 1 h. After cooling, the mixture was poured into H_2_O (100 mL) and extracted with EtOAc (3 × 50 mL). Then aqueous solution was treated with NaOH to pH 12 and extracted with CH_2_Cl_2_ (3 × 50 mL). The combined organic layer was washed with brine (2 × 100 mL), dried over Na_2_SO_4_, and evaporated. After flash column chromatography (EtOAc, then EtOH/NH_3_), compounds **15** and **16** were isolated as a mixture in ratio of 1:1 (brown oil, 300 mg, 57%).

#### 3.1.2. Preparation of Substituted 1-Benzoyl-β-Carbolines **18**, **19**, **23**, **24**

Corresponding acetophenone (0.458 mmol) and iodine (92 mg, 0.366 mmol) were added to 2 mL of DMSO, and the resulting solution was heated at 90 °C for 1 h. After that tryptamine, its derivative or their mixture (0.458 mmol) was added to the solution and this solution was stirred at the same temperature for 3–4 h till completion of reaction (monitored by TLC). Then the reaction mixture was cooled to room temperature followed by the addition of water (50 mL) and extraction with EtOAc (2 × 25 mL). The extract was washed with 10% Na_2_S_2_O_3_, dried over Na_2_SO_4_, filtered and evaporated under reduced pressure. The residue was purified by MPLC using benzene and benzene/hexanes as eluent to give the desired product.

For 1-(2,4-dibromobenzoyl)-7-bromo-β-carboline (**18**): yellow solid, 20%. ^1^H-NMR (400 MHz, CDCl_3_): δ 10.45 (br. s, 1H), 8.57 (d, *J* = 4.9 Hz, 1H), 8.15 (d, *J* = 4.9 Hz, 1H), 8.05 (d, *J* = 8.3 Hz, 1H), 7.88 (d, *J* = 1.7 Hz, 1H), 7.79 (d, *J* = 1.1 Hz, 1H), 7.62 (dd, *J* = 8.3, 1.7 Hz, 1H), 7.50 (dd, *J* = 8.3, 1.5 Hz, 1H), 7.46 (d, *J* = 8.2 Hz, 1H). ^13^C-NMR (100 MHz, CDCl_3_): δ 196.7, 141.4, 138.9, 138.4, 136.5, 135.2, 134.7, 130.9, 130.6, 129.8, 124.4, 124.2, 122.9, 122.6, 120.5, 119.2, 118.9, 114.8. HRMS-ESI, *m*/*z*: [M + H]^+^ calculated for C_18_H_10_^79^Br_3_N_2_O^+^: 506.8340, found 506.8345.

For 1-(2,4-dibromobenzoyl)-5-bromo-β-carboline (**19**): yellow solid, 19%. ^1^H-NMR (400 MHz, CDCl_3_): δ 10.55 (br. s, 1H), 8.81 (d, *J* = 5.1 Hz, 1H), 8.62 (d, *J* = 5.1 Hz, 1H), 7.88 (d, *J* = 1.8 Hz, 1H), 7.63 (dd, *J* = 3.2, 1.4 Hz, 1H), 7.61 (dd, *J* = 2.9, 1.4 Hz, 1H), 7.54–7.58 (m, 1H), 7.51 (d, *J* = 7.80 Hz, 1H), 7.46 (d, *J* = 8.2 Hz, 1H). ^13^C-NMR (100 MHz, CDCl_3_): δ 197.5, 142.4, 142.4, 139.4, 139.1, 137.0, 135.9, 135.1, 131.6, 131.3, 130.4, 130.2, 125.4, 125.1, 121.5, 121.2, 118.5, 111.3. HRMS-ESI, *m*/*z*: [M + H]^+^ calculated for C_18_H_10_^79^Br_3_N_2_O^+^ 506.8340, found 506.8347.

For 1-(2,4-dibromobenzoyl)-β-carboline (**23**): yellow solid, 38%. ^1^H-NMR (400 MHz, DMSO-d6): δ 12.23 (br. s, 1H, NH), 8.48 (d, *J* = 4.9, 1H, H-3), 8.44 (d, *J* = 4.9, 1H, H-4), 8.34 (d, *J* = 7.9, 1H, H-5), 8.02 (d, *J* = 1.9, 1H, H-3′), 7.85 (d, *J* = 8.0, 1H, H-8), 7.76 (dd, *J* = 8.3, 1.9, 1H, H-5′), 7.64 (ddd, *J* = 7.2, 7.2, 1.0, 1H, H-7), 7.57 (d, *J* = 8.3, 1H, H-6′), 7.35 (ddd, *J* = 7.2, 7.2, 1.0, 1H, H-6). ^13^C-NMR (100 MHz, DMSO-d6): δ 195.9, 142.0, 140.5, 137.9, 135.3, 134.8, 134.3, 131.4, 131.0, 130.3, 129.2, 128.3, 123.2, 121.9, 120.5, 120.0, 119.8, 113.1. HRMS-ESI, *m*/*z*: [M + H]^+^ calculated for C_18_H_11_^79^Br_2_N_2_O^+^ 428.9235, found 428.9239.

For 1-(2,5-dichlorobenzoyl)-β-carboline (**24**): yellow solid, 44%. ^1^H-NMR (400 MHz, CDCl_3_): δ 10.38 (br. s, 1H), 8.57 (d, *J* = 4.9 Hz, 1H), 8.19 (m, 2H), 7.65 (m, 2H), 7.57 (t, *J* = 1.4 Hz, 1H), 7.43 (d, *J* = 1.1 Hz, 2H), 7.39 (m, 1H). ^13^C-NMR (100 MHz, CDCl_3_): δ 196.1, 141.2, 139.6, 139.0, 136.8, 134.9, 132.5, 132.0, 131.1, 131.1, 130.1, 129.7, 129.6, 122.0, 121.2, 120.7, 119.5, 112.2. HRMS-ESI, *m*/*z*: [M + H]^+^ calculated for C_18_H_11_^35^Cl_2_N_2_O^+^ 341.0247, found 341.0242.

#### 3.1.3. Preparation of Fascaplysin Derivatives

Substituted 1-benzoyl-β-carboline (0.326 mmol) was heated in sealed tube at 220 °C for 15 min. After cooling, the reaction mixture was washed with EtOAc (3 × 3 mL) and H_2_O (3 × 10 mL). The combined aqueous layer was acidified with hydrochloric acid and evaporated under reduced pressure to give target product as a red powder.

For 3,10-dibromofascaplysin (**5**): red solid, 91%. ^1^H NMR (400 MHz, MeOH-d4): δ 9.38 (d, *J* = 6.4, 1H, H-6), 8.97 (d, *J* = 6.4, 1H, H-7), 8.69 (bs, 1H, H-4), 8,41 (d, *J* = 8.8, 1H, H-8), 8.05 (d, *J* = 1.4, 1H, H-11), 7.97 (d, *J* = 0.8 × 2, 2H, H-1, H-2), 7.71 (dd, *J* = 8.6, 1.7, 1H, H-9). ^13^C-NMR (100 MHz, MeOH-d4): δ 180.2, 147.7, 147.6, 140.8, 134.0, 131.4, 130.7, 128.7, 126.6, 126.4, 126.0, 124.8, 122.7, 119.5, 119.5, 118.7, 118.4, 115.9. ^13^C-NMR (100 MHz, DMSO-d6): δ 181.3, 148.0, 147.8, 140.2, 134.4, 131.2, 130.5, 128.2, 127.7, 127.1, 126.6, 126.1, 123.5, 123.3, 120.8, 119.6, 118.6, 116.4. HRMS-ESI, *m*/*z*: [M]^+^ calculated for C_18_H_9_^79^Br_2_N_2_O^+^ 426.9079, found 426.9085.

For compound **20**: red solid, 93%. ^1^H-NMR (400 MHz, MeOH-d4): δ 9.44 (d, *J* = 4.7 Hz, 1 H), 9.36 (d, *J* = 4.5 Hz, 1 H), 8.76 (s, 1 H), 7.98 (s, 2 H), 7.76–7.88 (m, 3 H). ^13^C-NMR (100 MHz, MeOH-d4): δ 180.1, 148.1, 147.5, 140.1, 134.5, 134.2, 131.4, 130.7, 126.8, 126.0, 122.7, 122.4, 120.3, 119.5, 118.9, 118.9, 118.5, 112.2. HRMS-ESI, *m*/*z*: [M]^+^ calculated for C_18_H_9_^79^Br_2_N_2_O^+^ 426.9079, found 426.9083.

For 3-bromofascaplysin (**3**): red solid, 84%. ^1^H-NMR (400 MHz, MeOH-d4): δ 9.35 (d, *J* = 6.2, 1H, H-6), 8.95 (d, *J* = 6.2, 1H, H-7), 8.68 (s, 1H, H-4), 8.48 (d, *J* = 8.1, 1H, H-8), 7.93 (s, 2H, H-1, H-2), 7.88 (t, *J* = 7.6, 1H, H-10), 7.79 (d, *J* = 8.1, 1H, H-11), 7.52 (t, *J* = 7.6, 1H, H-9). ^13^C-NMR (100 MHz, MeOH-d4): δ 182.0, 149.4, 148.9, 143.1, 136.0, 135.6, 132.3, 132.2, 127.7, 127.6, 125.1, 124.5, 124.5, 123.8, 121.1, 120.9, 120.3, 114.5. HRMS-ESI, *m*/*z*: [M]^+^ calculated for C_18_H_10_^79^BrN_2_O^+^ 348.9974, found 348.9980.

For compound **25**: red solid, 70%. ^1^H-NMR (400 MHz, MeOH-d4): δ 9.36 (d, *J* = 5.8, 1H), 8.96 (d, *J* = 6.0, 1H), 8.49 (d, *J* = 8.0, 1H), 8.35 (d, *J* = 8.6, 1H), 8.05 (d, *J* = 1.2, 1H), 7.98 (d, *J* = 7.5, 1H), 7.90 (d, *J* = 6.8, 1H), 7.82 (m, 1H), 7.55 (t, *J* = 7.6, 1H). ^13^C-NMR (100 MHz, MeOH-d4): δ 180.3, 147.2, 145.3, 136.7, 135.8, 134.3, 127.6, 126.5, 126.2, 126.1, 125.8, 124.9, 123.6, 123.0, 121.9, 119.5, 116.3, 112.9. HRMS-ESI, *m*/*z*: [M]^+^ calculated for C_18_H_10_^35^ClN_2_O^+^ 305.0480, found 305.0486.

#### 3.1.4. Preparation of Compounds **10**, **27**

A solution of compound **3** or **5** (0.15 mmol) in DMF (10 mL) was treated with solution of NaOH (24 mg, 0.6 mmol) in 0.1 mL of H_2_O at room temperature for 0.5 h. The mixture was neutralized with AcOH and evaporated under reduced pressure. The residue was washed with Et_2_O and dried.

For 14-bromoreticulatate (**10**): yellow solid, 86%. ^1^H-NMR (400 MHz, MeOH-d4): δ 9.37 (s, 1H), 8.75 (d, *J* = 6.3, 1H), 8.59 (d, *J* = 6.5, 1H), 8.47 (d, *J* = 8.1, 1H), 8.17 (d, *J* = 8.4, 1H), 8.06 (s, 1H), 8.00 (d, *J* = 8.4, 1H), 7.83 (m, 2H), 7.50 (t, *J* = 7.4, 1H). ^13^C-NMR (100 MHz, MeOH-d4): δ 152.1, 144.6, 142.4, 134.2, 133.4, 133.4, 133.1, 132.1, 131.9, 130.0, 129.1, 123.0, 122.7, 121.5, 119.5, 119.0, 116.0, 112.1. HRMS-ESI, *m*/*z*: [M]^+^ calculated for C_18_H_12_^79^BrN_2_O_2_^+^ 367.0079, found 367.0084.

For compound **27**: insoluble in most solvents ivory solid, 80%. It was introduced into next step without further purification.

#### 3.1.5. Preparation of 14-Bromoreticulatine (**7**) and Compound **28**

A mixture of compound **10** or **27** (0.08 mmol), acetonitrile (1 mL), sodium carbonate (0.57 mmol) and dimethyl sulfate (0.32 mmol) was stirred at room temperature for 0.5 h. The mixture was evaporated under reduced pressure and washed with H_2_O (3 mL). The resulting oil was triturated with Et_2_O and dried.

For 14-bromoreticulatine (**7**): yellow solid, 52%. ^1^H-NMR (400 MHz, MeOH-d4): δ 9.40 (s, 1H), 8.76 (d, *J* = 6.4 Hz, 1H), 8.60 (d, *J* = 6.4 Hz, 1H), 8.48 (d, *J* = 8.1 Hz, 1H), 8.20 (d, *J* = 8.5 Hz, 1H), 8.12 (d, *J* = 1.9 Hz, 1H), 8.05 (dd, *J* = 8.5, 1.8 Hz, 1H), 7.79–7.87 (m, 2H), 7.50 (ddd, *J* = 7.5, 7.5, 0.9 Hz, 1H), 3.64 (s, 3H). ^13^C-NMR (100 MHz, MeOH-d4): δ 162.8, 144.8, 143.2, 134.2, 134.2, 133.7, 133.1, 132.8, 132.4, 130.8, 130.0, 127.1, 124.5, 122.8, 121.7, 119.0, 116.1, 112.3, 51.5. HRMS-ESI, *m*/*z*: [M]^+^ calculated for C_19_H_14_^79^BrN_2_O_2_^+^ 381.0235, found 381.0242.

For compound **28**: yellow solid 48%. ^1^H-NMR (400 MHz, MeOH-d4): δ 8.90 (br. s, 1H), 8.43 (d, *J* = 6.1 Hz, 1H), 8.20 (d, *J* = 8.7 Hz, 1H), 8.05 (d, *J* = 6.3 Hz, 1H), 7.91 (s, 1H), 7.77–7.88 (m, 3H), 7.26 (d, *J* = 8.4 Hz, 1H), 3.69 (s, 4 H), 3.35 (s, 3H). HRMS-ESI, *m*/*z*: [M]^+^ calculated for C_20_H_15_^79^Br_2_N_2_O_2_^+^ 473.0096, found 473.0103.

### 3.2. Biological Evaluation

McCoy’s 5A Medium (McCoy), Roswell Park Memorial Institute Medium (RPMI 1640), Dulbecco’s Modified Eagle Medium (DMEM), phosphate buffered saline (PBS), L-glutamine, penicillin–streptomycin solution, trypsin, fetal bovine serum (FBS), sodium hydrocarbonate (NaHCO_3_), and agar were purchased from “Biolot” (Russia).

#### 3.2.1. Cell Lines and Culture Conditions

Human colorectal carcinoma HT-29 (ATCC^®^ no. HTB-38™), human breast cancer T-47D (ATCC^®^ no. HTB-133™), and melanoma SK-MEL-28 (ATCC^®^ no. HTB-72™) cell lines were gifted by Hormel Institute University of Minnesota (Austin, MN, USA). Human prostate cancer PC-3 (ATCC^®^ no. CRL-1435™) and 22Rv1 (ATCC^®^ no. CRL-2505) cells were purchased from ATCC (Manassas, VA, USA). Human colorectal carcinoma HT-29, human breast cancer T-47D, and melanoma SK-MEL-28 cell lines were cultured in McCoy, RPMI-1640, and DMEM medium, respectively. Medium were supplemented with 5% and 10% fetal bovine serum (FBS), 200 mM L-glutamine, and penicillin-streptomycin solution. The cell cultures were maintained at 37 °C in humidified atmosphere containing 5% CO_2_. The human prostate cancer PC-3 and 22Rv1 cells were cultured according to the manufacturer’s instructions in RPMI media (Invitrogen), supplemented with GlutamaxTM-I (Invitrogen, Paisley, UK) and contained 10% FBS (Invitrogen) and 1% penicillin/streptomycin (Invitrogen). Cells were continuously kept in culture for a maximum of 3 months, and were routinely checked for contamination with mycoplasma and inspected microscopically for stable phenotype. Several test cultures were used to determine antibiotic activity, including *Bacillus subtilis* (KMM 430), *Staphylococcus aureus* (ATCC 21027), *P. aeruginosa* (KMM 433), *Escherichia coli* (ATCC 15034), and *Candida albicans* (KMM 455). All cultures are stored in the collection of marine microorganisms of the PIBOC FEB RAS, the official acronym of CMM [40]. Antibiotic activity was determined with the disk diffusion soft agar assay as described before [41].

#### 3.2.2. Cytotoxicity Assays

MTS and MTT assays were used as an indicator of cell viability as determined by mitochondrial-dependent reduction of formazan or its salts. For MTS assay, the cells were seeded in density of 1.0 × 10^4^ cells/200 µL of complete medium in 96-well plates. After incubation for 24 h attached cells were treated with various concentrations of the compounds (0.05; 0.1; 0.5; 1; 5 µM), while the control was treated with the complete McCoy, RPMI-1640, and DMEM medium only. Cells were cultured for additional 24 h at 37 °C in 5% CO_2_ incubator. After incubation, MTS-reagent (20 µL) was added to each well, and then cells were incubated for 3 h at 37 °C in 5% CO_2_. Absorbance was measured at 490/630 nm by microplate reader (Power Wave XS, American). All tested samples were carried out in triplicates. MTT assay was performed as previously described with the 48 h drug treatment [42]. The trypan-blue-based viability assay (ViCell assay) was performed using Beckman Coulter Vi-CELL (Beckman Coulter, Krefeld, Germany) as has been described before [43].

#### 3.2.3. Statistical Analysis

Statistical analyses were performed using GraphPad Prism software v. 5.01 (GraphPad Prism software Inc., La Jolla, CA, USA). Data are presented as mean ± SD. The unpaired Student’s *t*-test was used for the comparison of two groups. Statistical significance was represented as * *p* < 0.05 and ** *p* < 0.01.

## 4. Conclusions

Thus, the two-step approach toward the synthesis of the marine sponge derived pigment fascaplysin was used to obtain the marine alkaloids 3-bromofascaplysin and 3,10-dibromofascaplysin. These compounds were used as the starting materials for first syntheses of the alkaloids 14-bromoreticulatine and 14-bromoreticulatate. Preliminary bioassays showed that 14-bromoreticulatine reveals selective antibiotic (to *P. aeruginosa*) and cytotoxic (to melanoma SK-MEL-28 cell line) activities. It was also demonstrated that 3,10-dibromofascaplysin was able to suppress the cell metabolism at concentrations at least 7 times lower than the cytotoxic concentrations, which induced cell membrane disruption. The examination of biological activity of the synthesized compounds showed that even minimal modification of fascaplysin structure has a significant effect on the bioactivity of this lead compound. At the present time, the biological activities of a large series of novel synthetic derivatives of fascaplysin are being investigated thoroughly. This should open new opportunities for the detailed studies of the structure–activity relationships among these potent and promising biologically active substances. 

## Figures and Tables

**Table 1:** Cytotoxic activities of fascaplysin and its derivatives. Values are indicated as mean ± standard deviation.

Compound	Inhibiting Concentration, (IC_50_), µM						IC_50_ (ViCell)/IC_50_ (MTT) for 22Rv1 Cells
	HT-29 ^a^	T-47D ^a^	SK-MEL-28 ^a^	PC-3 ^b^	22Rv1 ^b^	22Rv1 ^c^	
Fascaplysin (**1**)	2.7 ± 0.05	5 ± 0.2	1.3 ± 0.08	0.78 ± 0.16	0.24 ± 0.04	0.34 ± 0.11	1.39
3-Bromofascaplysin (**3**)	3.3 ± 0.12	>5	1.9 ± 0.04	10 ± 1.75	0.42 ± 0.29	0.24 ± 0.14	0.58
Compound **20**	>5	>5	>5	1.39 ± 0.43	0.21 ± 0.04	0.26 ± 0.05	1.24
Compound **25**	>5	>5	1.8±0.02	0.91 ± 0.06	0.27 ± 0.01	0.5 ± 0.19	1.88
14-Bromoreticulatate (**10**)	>5	>5	>5	n/d	n/d	n/d	n/d
14-Bromoreticulatine (**7**)	>5	>5	1.2 ± 0.1	> 50	35.72 ± 10.1	n/d	n/d
3,10-Dibromofascaplysin (**5**)	>5	>5	>5	7.28 ± 0.73	0.69 ± 0.05	5.14 ± 1.16	7.45

IC_50_, the concentration of compounds that caused a 50% reduction in cell viability of tested normal and cancer cells; ^a^ MTS assay was used; ^b^ MTT assay was used; ^c^ ViCell assay (trypan blue exclusion) was used, n/d—not determined.

**Table 2:** Antibiotic activity of compounds **1**, **3**, **25**, and **7**.

Compound	Conc., mg/disc	Zone Unit Differentials in the Disk Diffusion Soft Agar Assay ^a^				
		*Bacillus subtilis* (KMM 430)	*Staphylococcus aureus* (ATCC 21027)	*Pseudomonas aeruginosa* (KMM 433)	*Escherichia coli* (ATCC 15034)	*Candida albicans* (KMM 455)
**1**	0.4	25	25	>35	20	n/a
**3**	0.1	25	20	>35	20	*
**25**	0.2	20	20	>35	20	n/a
**7**	0.2	10	n/a	>35	10	n/a

^a^ Measured in mm; * fungistatic effect; n/a, not active.

## Supplementary Materials

The following are available online at https://www.mdpi.com/1660-3397/17/9/496/s1, Comparison of ^1^H-NMR data of synthetic and natural 3-bromofascaplysin, 3.10-dibromofascaplysin, 14-bromoreticulatate and 14-bromoreticulatine. Spectra Data.
