# Supplementary material for: Total Syntheses and Preliminary Biological Evaluation of Brominated Fascaplysin and Reticulatine Alkaloids and Their Analogues
Source: Mar Drugs. 2019 Aug 25;17(9):496. doi: 10.3390/md17090496 (PMC6780422; doi:10.3390/md17090496)

Supporting Information

Total syntheses and bioactivities of brominated fascaplysin and reticulatine alkaloids and their analogues

Maxim E. Zhidkov,[1,*] Polina A. Smirnova,[1] Oleg A. Tryapkin,[1] Alexey V. Kantemirov,[1] Yulia V. Khudyakova,[2] Olesya S. Malyarenko,[2] Svetlana P. Ermakova,[2] Valeria P. Grigorchuk,[3] Moritz Kaune,[4] Gunhild von Amsberg[4,5] and Sergey A. Dyshlovoy[1,2,4,5]

[1] School of Natural Sciences, Far Eastern Federal University, 8 Sukhanov Str., Vladivostok 690950, Russian Federation

[2] G.B. Elyakov Pacific Institute of Bioorganic Chemistry, 159 Prospekt 100 Let Vladivostoku, Vladivostok 690022, Russian Federation

[3] Federal Scientific Center of the East Asia Terrestrial Biodiversity (Institute of Biology and Soil Science), Far Eastern Branch of the Russian Academy of Sciences, 159 Prospect 100-Let Vladivostoku, Vladivostok, 690022, Russian Federation

[4] Department of Oncology, Hematology and Bone Marrow Transplantation with Section Pneumology, Hubertus Wald-Tumorzentrum, University Medical Center Hamburg-Eppendorf, Hamburg, Germany

[5] Martini-Klinik Prostate Cancer Center, University Hospital Hamburg-Eppendorf, Hamburg, Germany

*Corresponding author: e-mail: zhidkov.me@dvfu.ru; phone: +79247353506

Contents

Comparison of 1H-NMR data of synthetic and natural 3-bromofascaplysin, 3.10-dibromofascaplysin, 14-bromoreticulatate and 14-bromoreticulatine …………..3-6

Spectra Data ………………………………………………………...……… 7-27

Comparison of ^{1}H-NMR data of synthetic and natural 3-bromofascaplysin, 3.10-dibromofascaplysin, 14-bromoreticulatate and 14-bromoreticulatine

1. 3-Bromofascaplysin (recorded in MeOH-d4)[1]

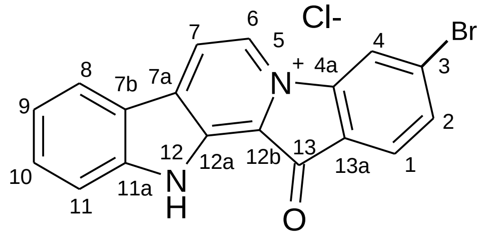

Position	^{1}H NMR	
	synthetic	natural
1	7.93, s	7.93, s
2	7.93, s	7.93, s
4	8.68 s	8.66, s
6	9.35, d (6.2)	9.34, d (6.0)
7	8.95, d (6.2)	8.93, d (6.0)
8	8.48, d (8.1)	8.45, d (7.5)
9	7.52, t (7.6)	7.51, t (7.5)
10	7.88, t (7.6)	7.87, t (7.5)
11	7.79, d (8.1)	7.76, d (7.5)

[1] NMR data of natural 3-bromofascaplysin presented in Segraves, N. L.; Lopez, S.; Johnson, T. A.; Said, S. A.; Fu, X.; Schmitz, F. J.; Pietraszkiewicz, H.; Valeriotec, F. A.; Crews, P. Structures and cytotoxicities of fascaplysin and related alkaloids from two marine phyla—*Fascaplysinopsis* sponges and *Didemnum* tunicates. *Tetrahedron Lett.* **2003**, *44,* 3471-3475.

2. 3.10-Dibromofascaplysin (recorded in MeOH-d4)[2]

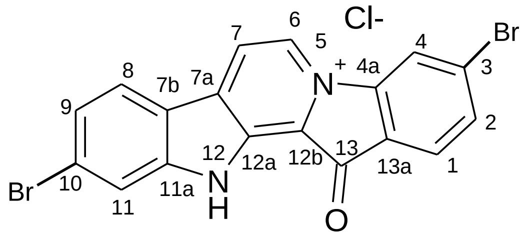

Position	^{1}H NMR	
	synthetic	natural
1	7.97, d (0.8)	7.96, d (1.2)
2	7.97, d (0.8)	7.96, d (1.2)
4	8.69, bs	8.70, t (0.9)
6	9.38, d (6.4)	9.39, d (6.4)
7	8.97, d (6.4)	8.97, d (6.4)
8	8.41, d (8.8)	8.41, d (8.4)
9	7.71, dd (8.6, 1.7)	7.70, dd (8.5, 1.6)
11	8.05, d (1.4)	8.03, d (1.2)

[2] NMR data of natural 3.10-dibromofascaplysin presented in Segraves, N. L.; Robinson, S. J.; Garcia, D. Comparison of Fascaplysin and Related Alkaloids: A Study of Structures, Cytotoxicities, and Sources *J. Nat. Prod.* **2004**, *67*, 783–792.

3. 14-Bromoreticulatate (recorded in MeOH-d4)[3]

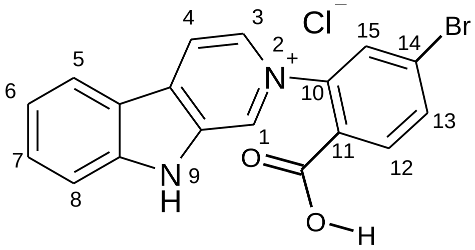

Position	^{1}H NMR		
	14-Bromoreticulatate ***(10)***	*14-Bromoreticulatate* ***(10)*** *(dried)*	natural
1	9.29, s	9.37, s	9.41, bs
3	8.56, d (6.4)	8.59, d (6.5)	8.63, dd (6.4, 1.2)
4	8.72, d (6.4)	8.75, d (6.3)	8.77, d (6.6)
5	8.46, d (8.1)	8.47, d (8.1)	8.50, ddd (8.1, 0.9, 0.9)
6	7.50, t (7.4)	7.50, t (7.4)	7.53, ddd (8.1, 7.0, 1.0)
7	7.85, m	7.83, m	7.88, ddd (8.4, 7.1, 1.3)
8	7.78, m	7.83, m	7.82, ddd (8.4, 0.8, 0.8)
12	8.01, m	8.17, d (8.4)	8.25, d (8.4)
13	7.93, dd (8.2, 1.5)	8.00, d (8.4)	8.07, dd (8.5, 1.9)
15	7.98, d (1.7)	8.06, s	8.12, d (1.7)

[3] NMR data of natural 14-bromoreticulatate presented in Segraves, N. L.; Lopez, S.; Johnson, T. A.; Said, S. A.; Fu, X.; Schmitz, F. J.; Pietraszkiewicz, H.; Valeriotec, F. A.; Crews, P. Structures and cytotoxicities of fascaplysin and related alkaloids from two marine phyla—*Fascaplysinopsis* sponges and *Didemnum* tunicates. *Tetrahedron Lett.* **2003**, *44,* 3471-3475.

4. 14-Bromoreticulatine (recorded in MeOH-d4)[4]

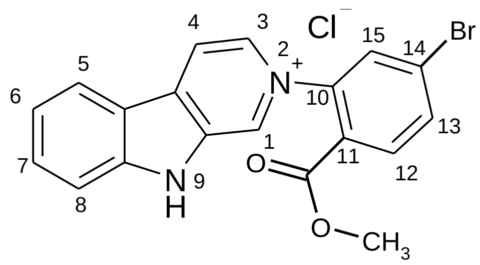

Position	^{1}H NMR	
	synthetic	natural
1	9.40, s	9.41, bs
3	8.60, d (6.4)	8.60, d (6.4)
4	8.76, d (6.4)	8.76, d (6.3)
5	8.48, d (8.1)	8.48, d (8.1)
6	7.50, dt (7.5, 0.9)	7.51, t (7.4)
7	7.84, m	7.84, m
8	7.84, m	7.84, m
12	8.20, d (8.5)	8.21, d (8.3)
13	8.05, dd (8.5, 1.8)	8.06, dd (8.4, 1.6)
15	8.12, d (1.9)	8.13, d (1.6)
OCH_3	3.64, s	3.66, s

[4] NMR data of natural 14-bromoreticulatine presented in Segraves, N. L.; Lopez, S.; Johnson, T. A.; Said, S. A.; Fu, X.; Schmitz, F. J.; Pietraszkiewicz, H.; Valeriotec, F. A.; Crews, P. Structures and cytotoxicities of fascaplysin and related alkaloids from two marine phyla—*Fascaplysinopsis* sponges and *Didemnum* tunicates. *Tetrahedron Lett.* **2003**, *44,* 3471-3475

Spectra Data

^{1}H NMR spectra of 1-(2.4-dibromobenzoyl)-7-bromo-β-carboline (18).

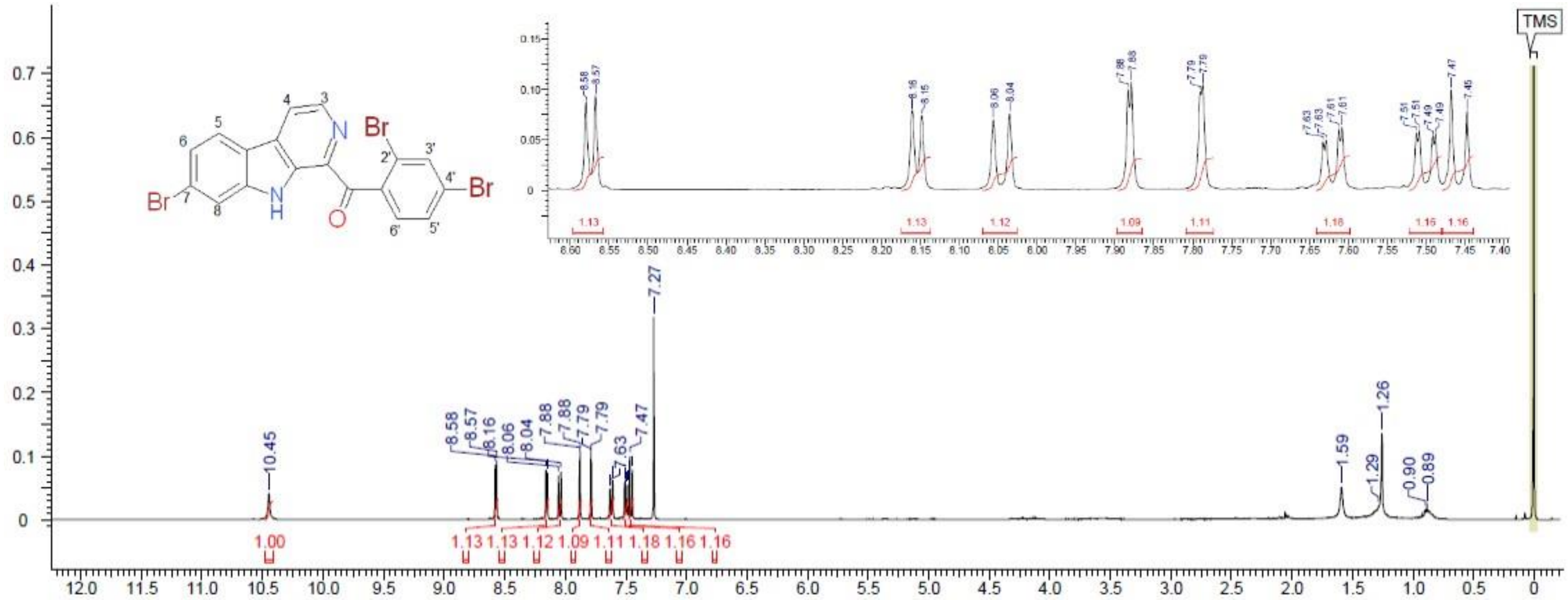

^{13}C NMR spectra of 1-(2.4-dibromobenzoyl)-7-bromo-β-carboline (18).

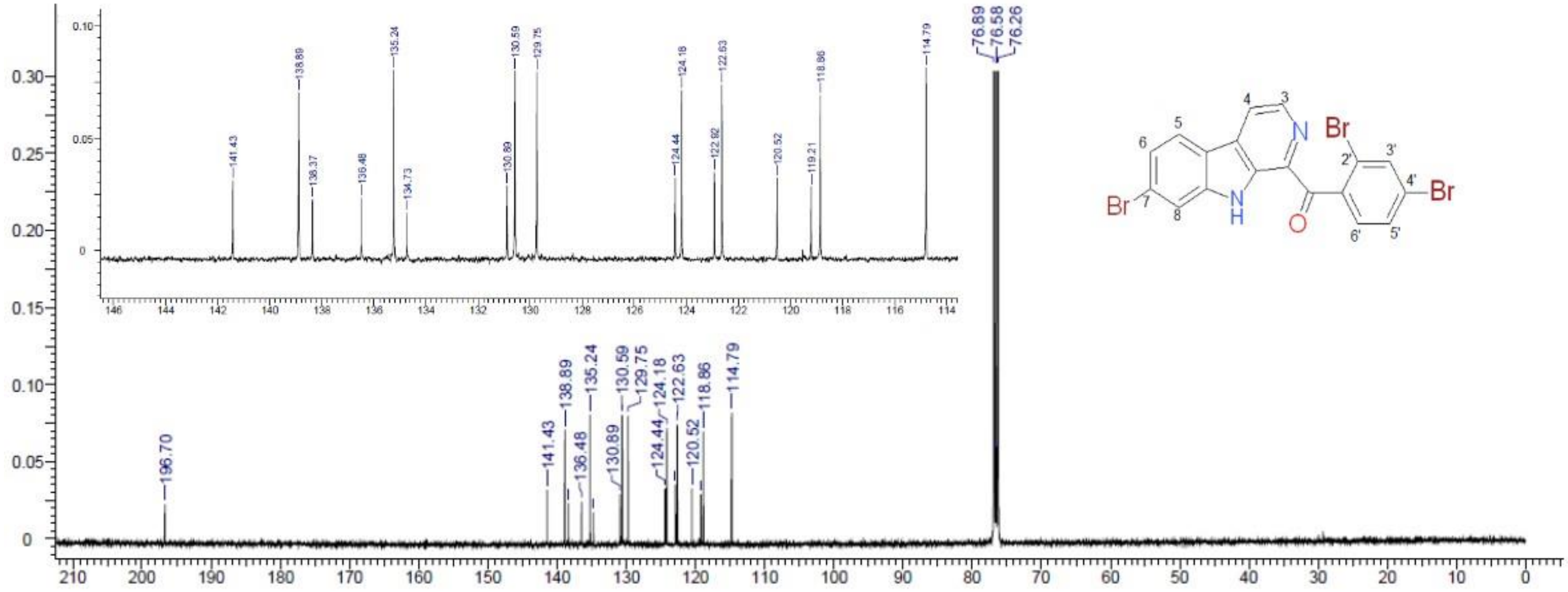

^{1}H NMR spectra of 1-(2.4-dibromobenzoyl)-5-bromo-β-carboline (19).

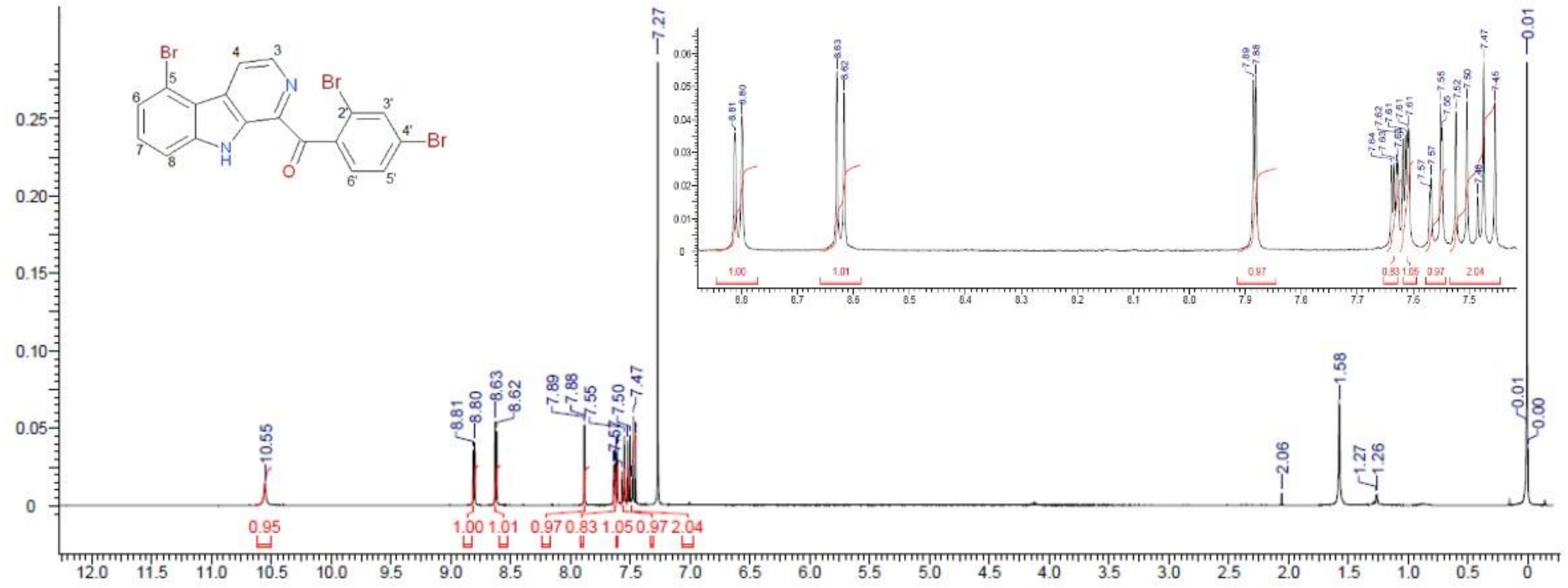

^{13}C NMR spectra of 1-(2.4-dibromobenzoyl)-5-bromo-β-carboline (19).

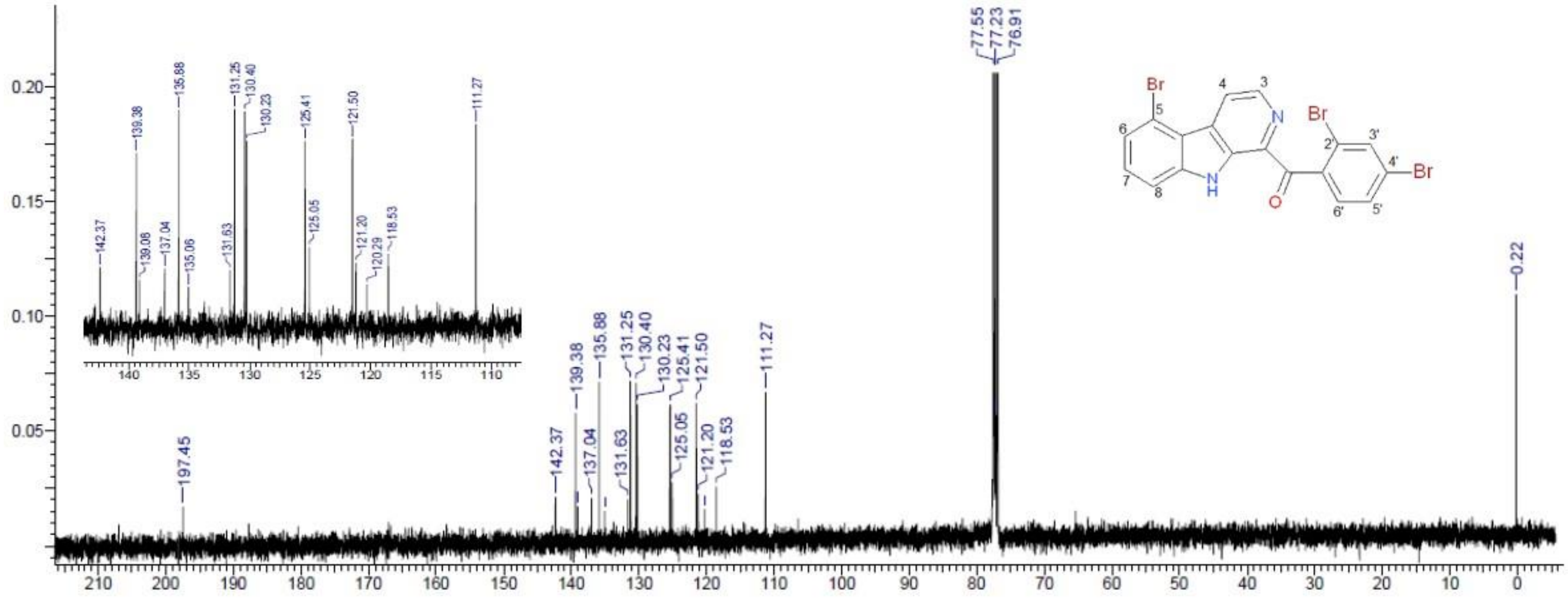

^{1}H NMR spectra of 1-(2.4-dibromobenzoyl)-β-carboline.

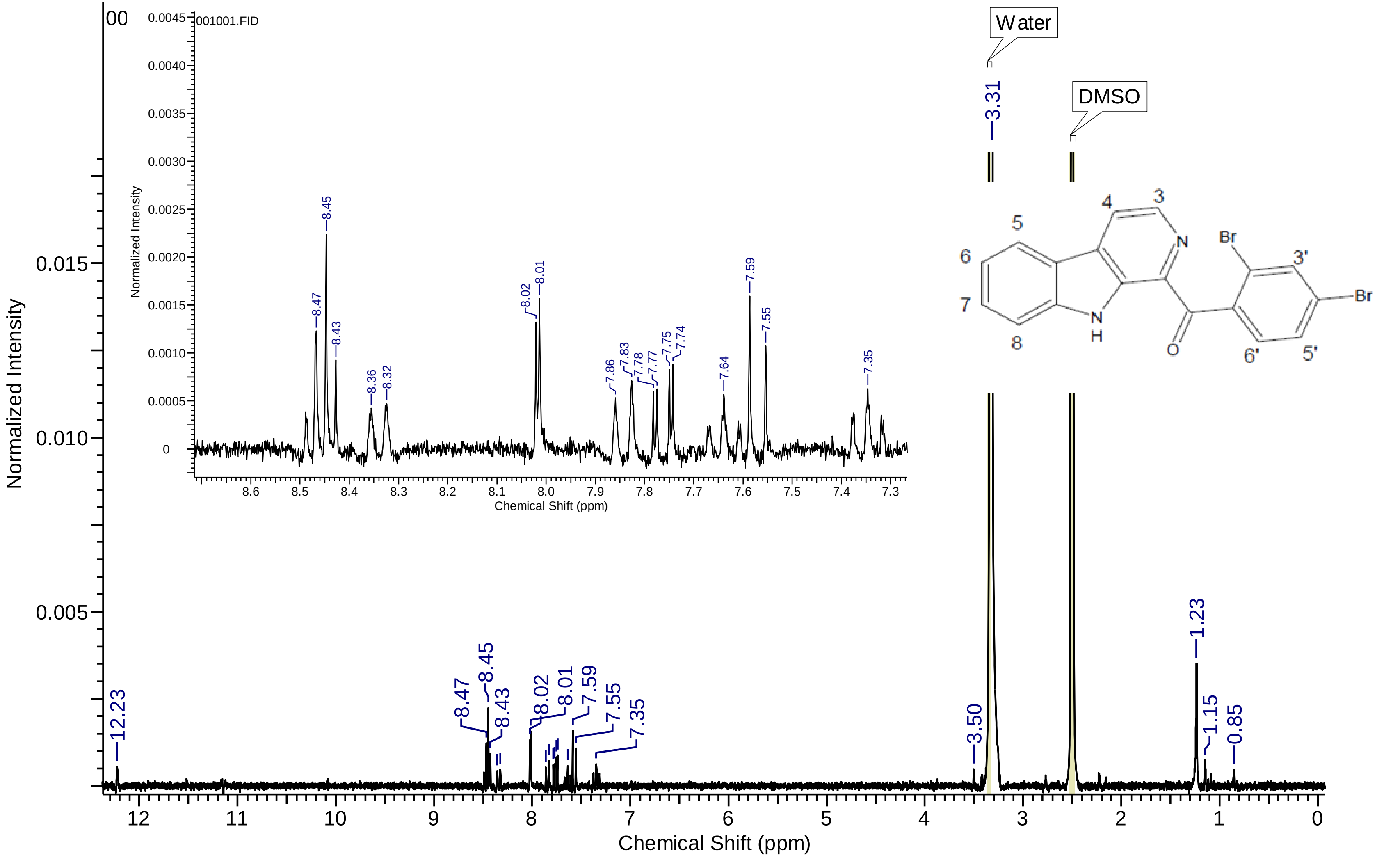

^{13}C NMR spectra of 1-(2.4-dibromobenzoyl)-β-carboline.

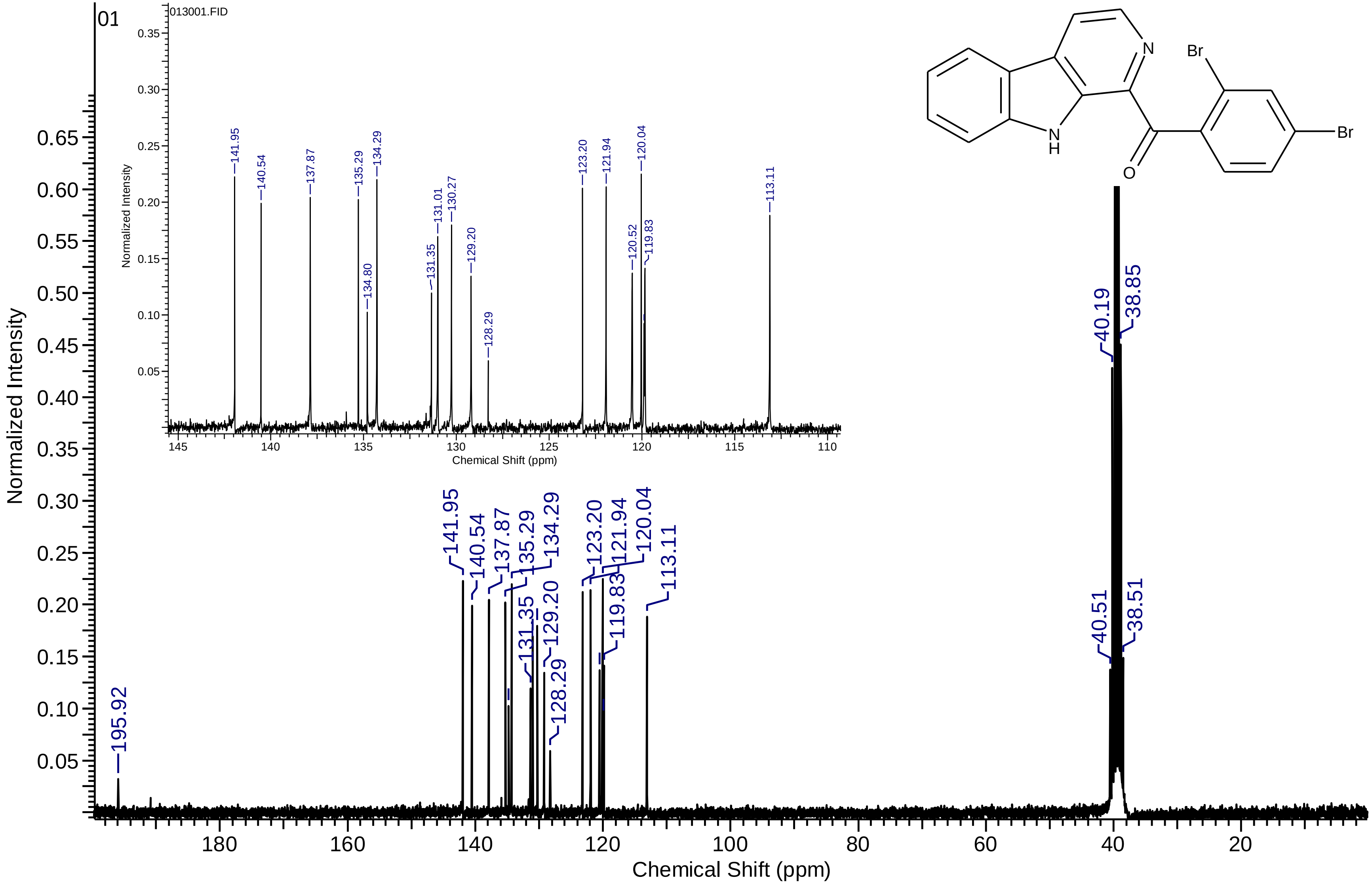

^{1}H NMR spectra of 1-(2.5-dichlorobenzoyl)-β-carboline.

Acquisition Time (sec)	5.1249	*Comment*	1H 16ppm	*Date*	18 Oct 2017 14:56:48		
Date Stamp	18 Oct 2017 14:56:48						
Frequency (MHz)	400.13	*Nucleus*	1H	*Number of Transients*	117	*Origin*	spect
Original Points Count	32768	*Owner*	nmr	*Points Count*	65536	*Pulse Sequence*	zg30
Receiver Gain	406.00	*SW(cyclical) (Hz)*	6393.86	*Solvent*	CHLOROFORM-d	*Spectrum Offset (Hz)*	3109.9343
Spectrum Type	STANDARD	*Sweep Width (Hz)*	6393.76	*Temperature (degree C)*	18.400		

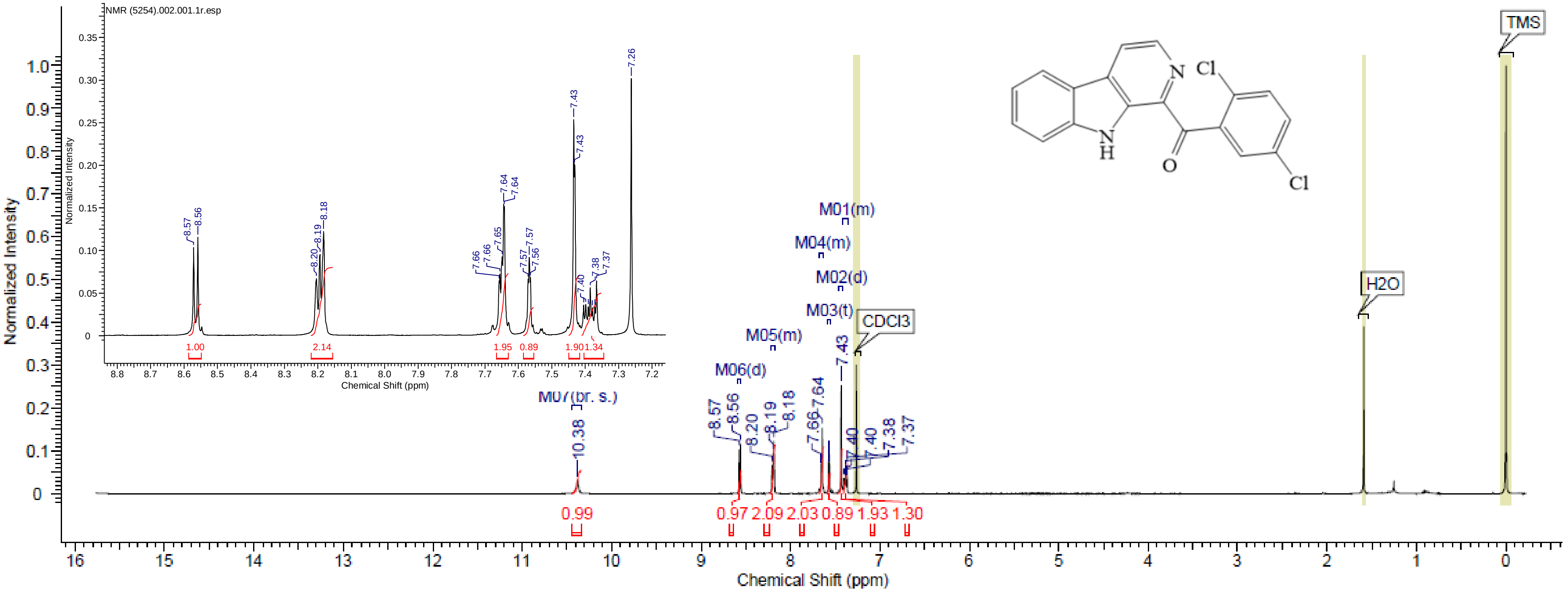

No.	Shift1 (ppm	H's	Type	J (Hz)	Multiplet1	(ppm)
1	7.39	1	m	-	M01	[7.36 .. 7.41]
2	7.43	2	d	1.07	M02	[7.42 .. 7.46]
3	7.57	1	t	1.40	M03	[7.55 .. 7.59]
4	7.65	2	m	-	M04	[7.63 .. 7.68]
5	8.19	2	m	-	M05	[8.17 .. 8.23]
6	8.57	1	d	4.88	M06	[8.55 .. 8.59]
7	10.38	1	br. s.	-	M07	[10.34 .. 10.45]

^{13}C NMR spectra of 1-(2.5-dichlorobenzoyl)-β-carboline.

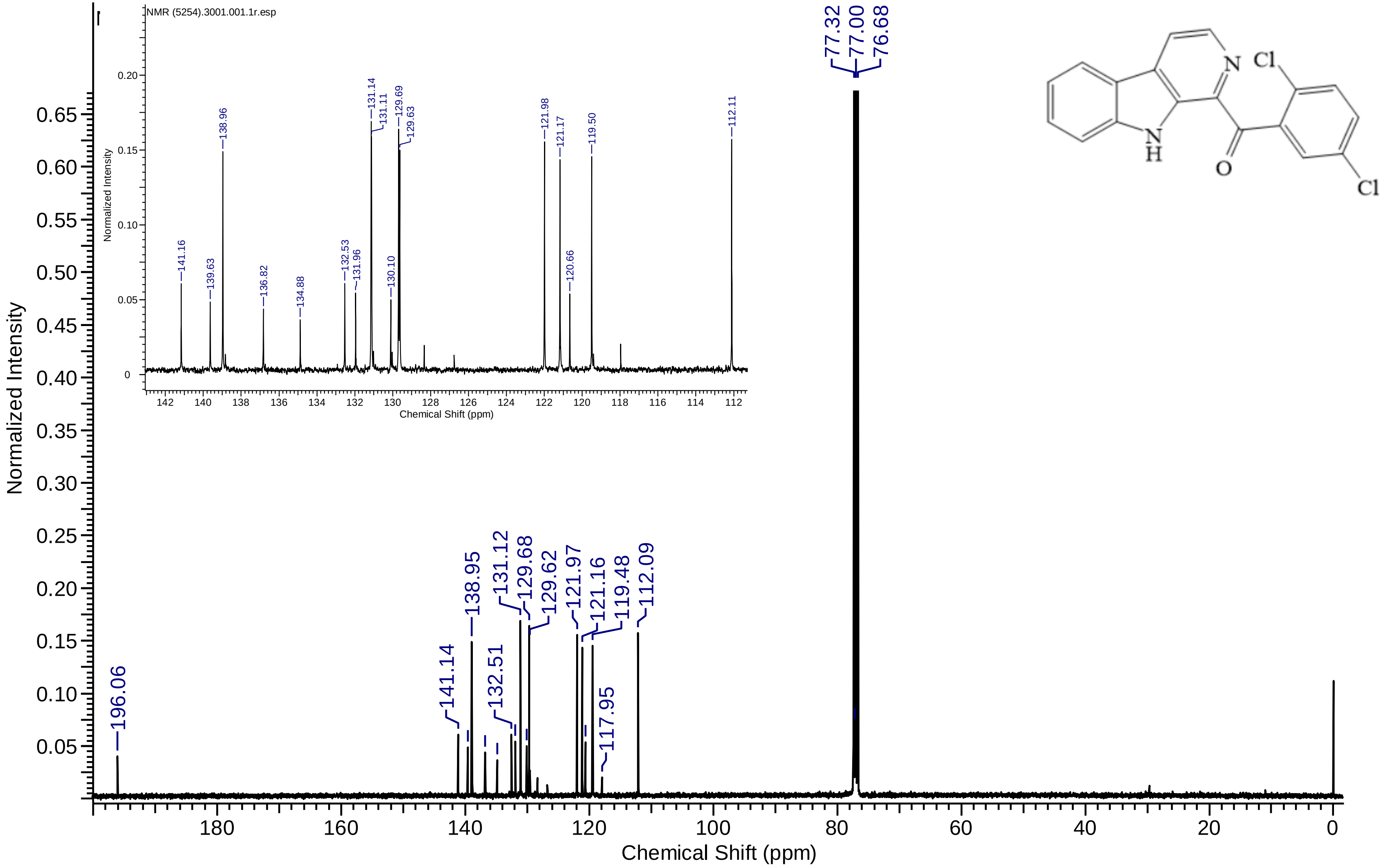

^{1}H NMR spectra of 3.10-dibromofascaplysin (5)

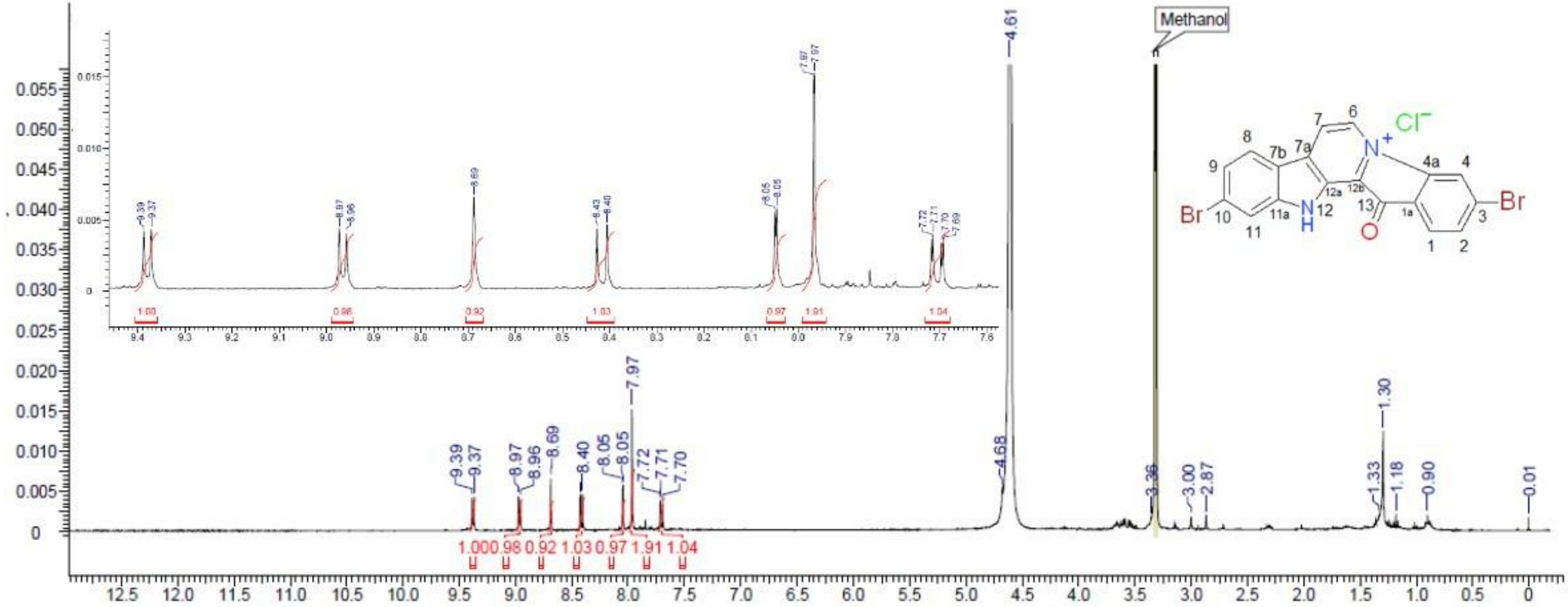

^{13}C NMR spectra of 3.10-dibromofascaplysin (5)

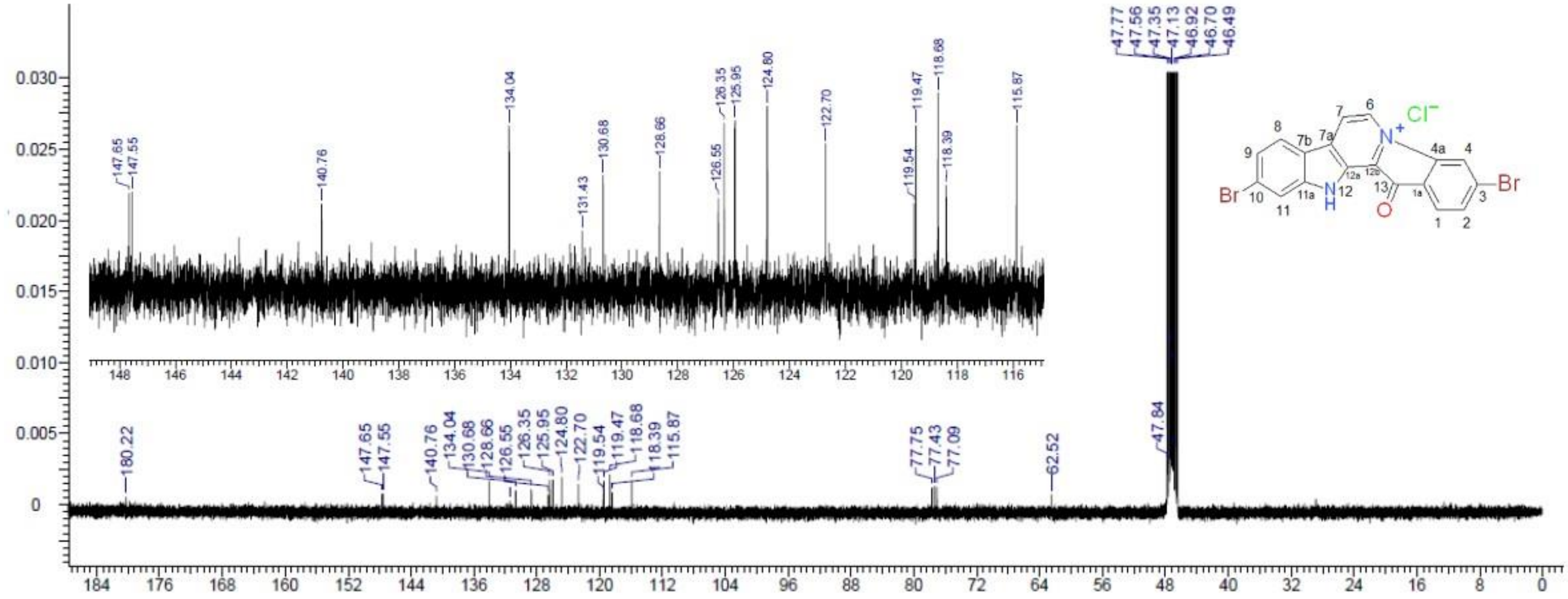

^{1}H NMR spectra of compound 20

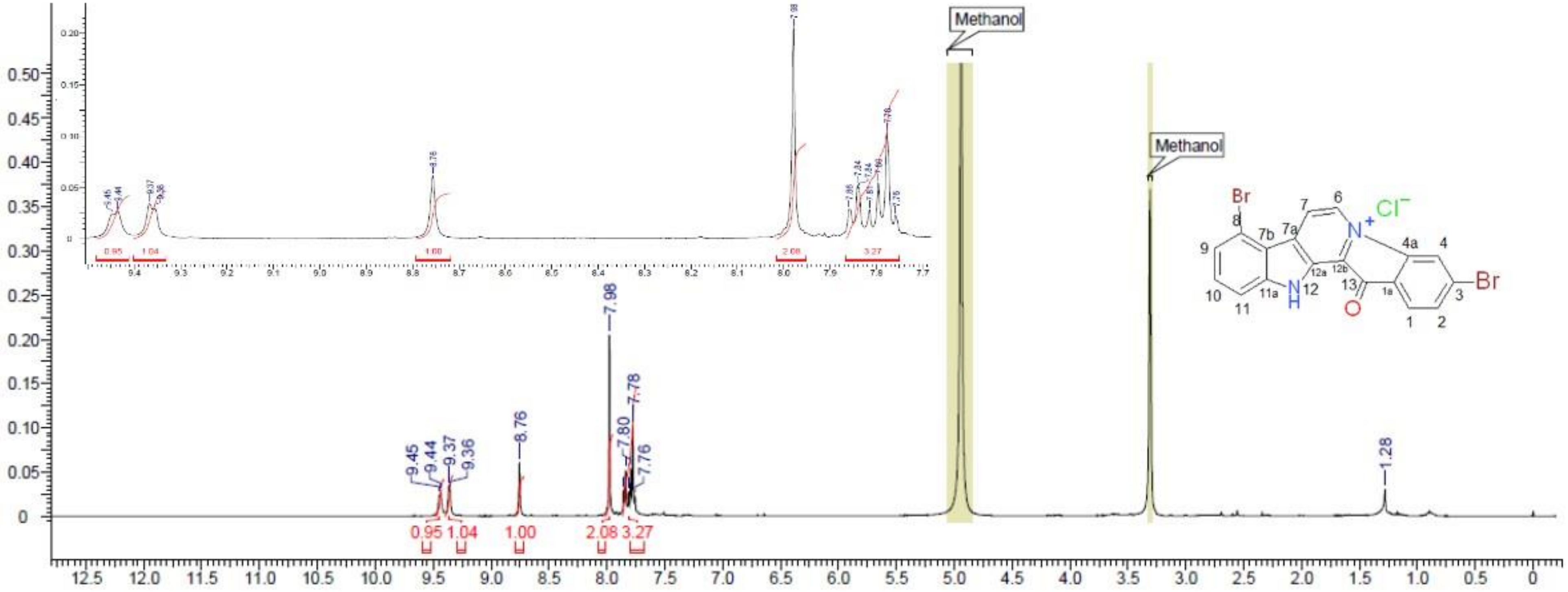

^{13}C NMR spectra of compound 20

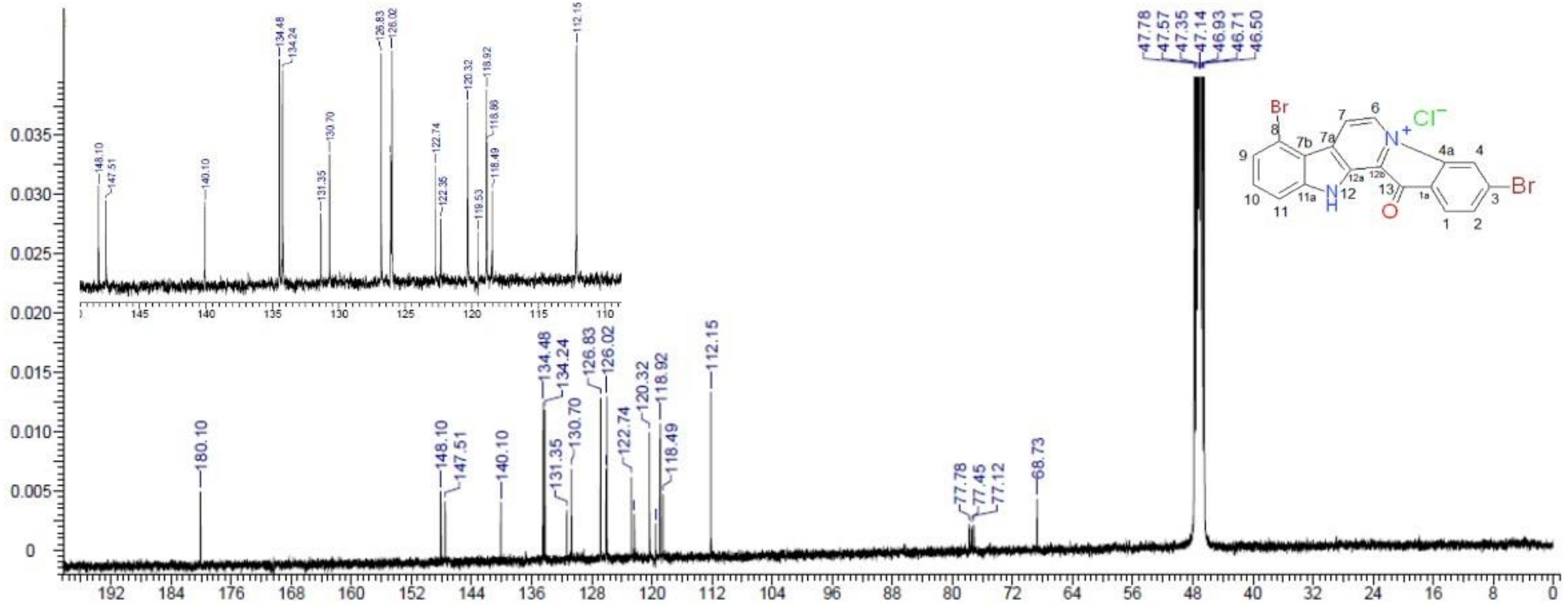

^{1}H NMR spectra of 3-bromofascaplysin (3)

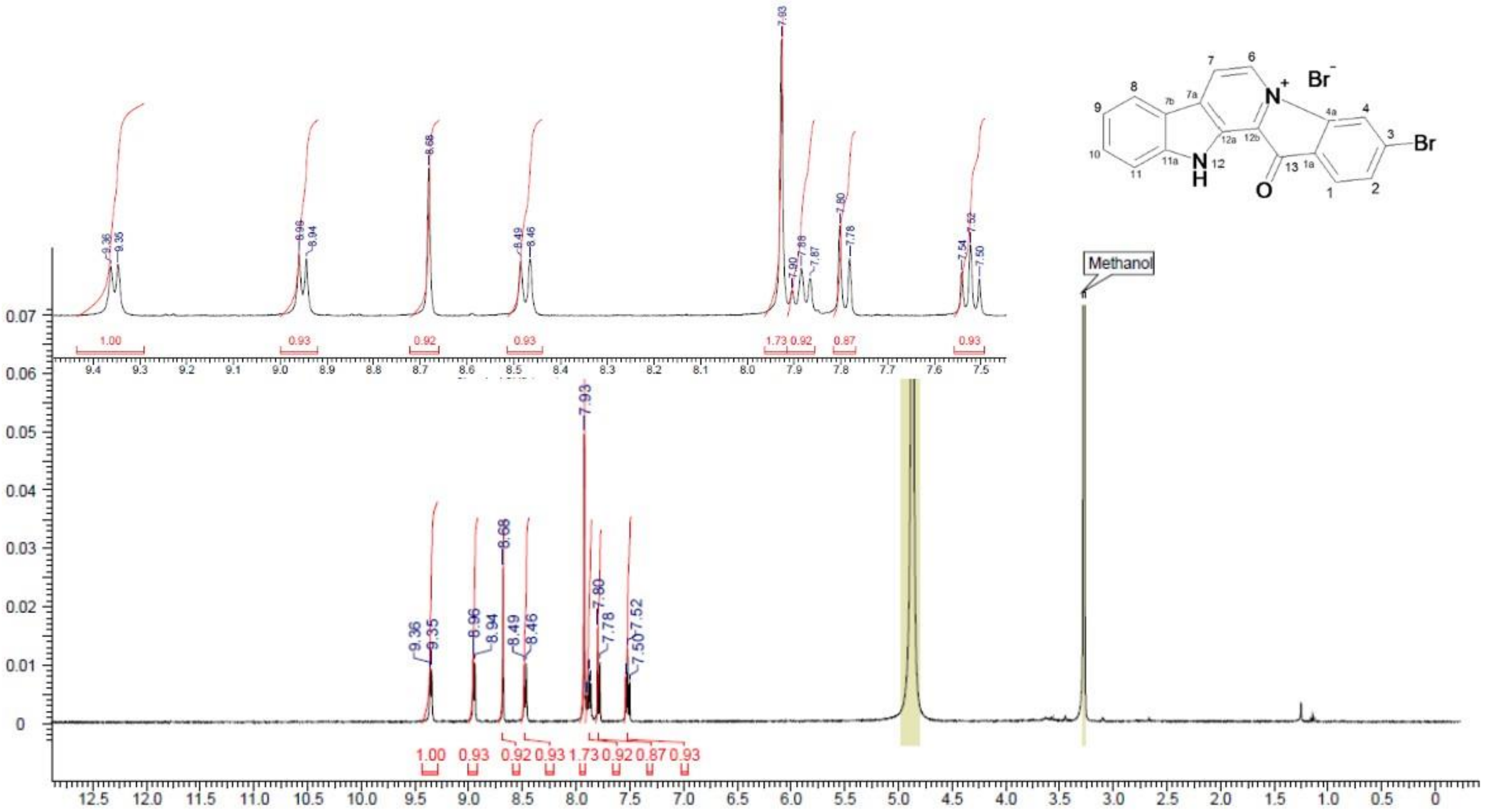

^{13}C NMR spectra of 3-bromofascaplysin (3)

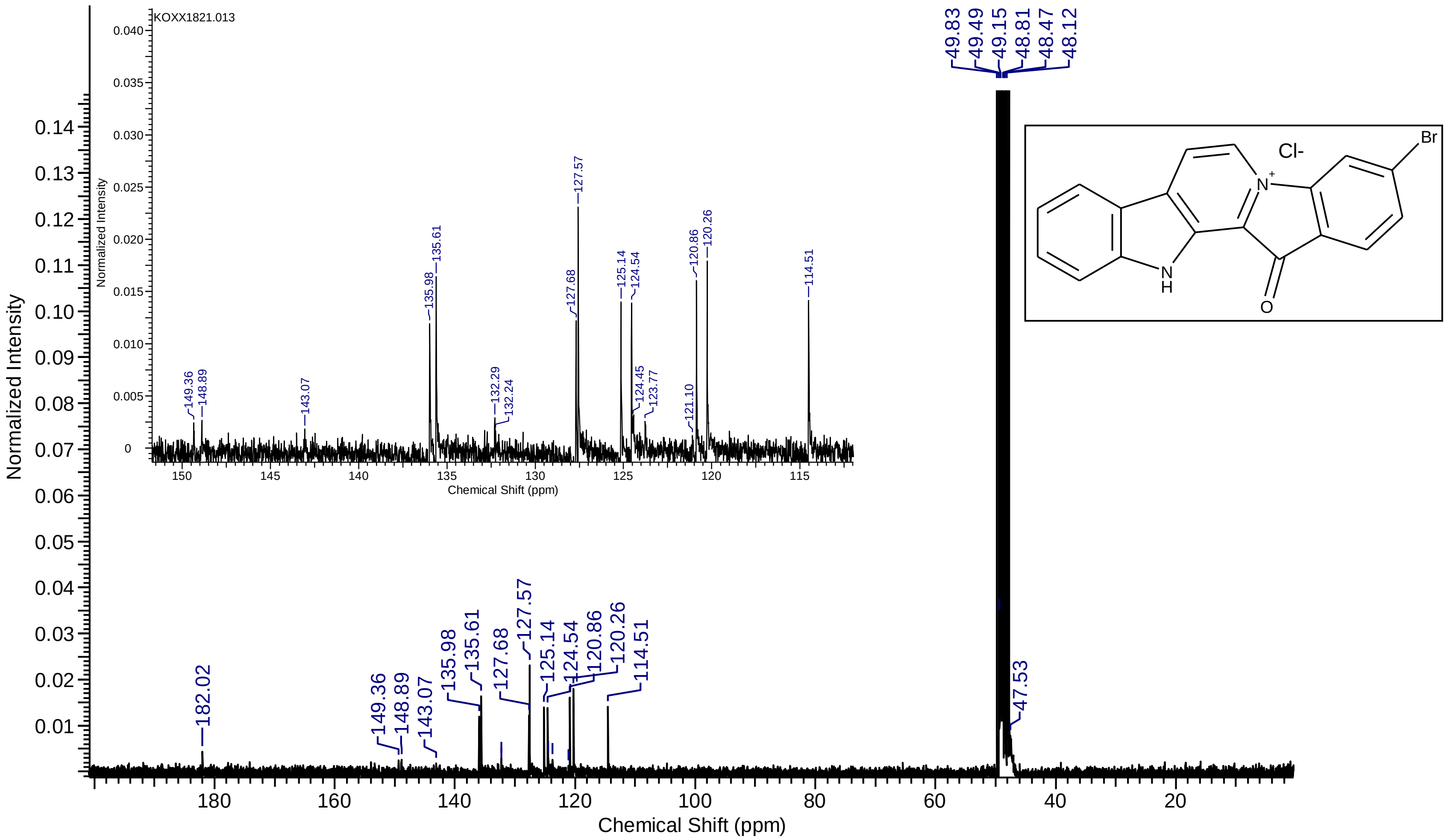

^{1}H NMR spectra of compound 21

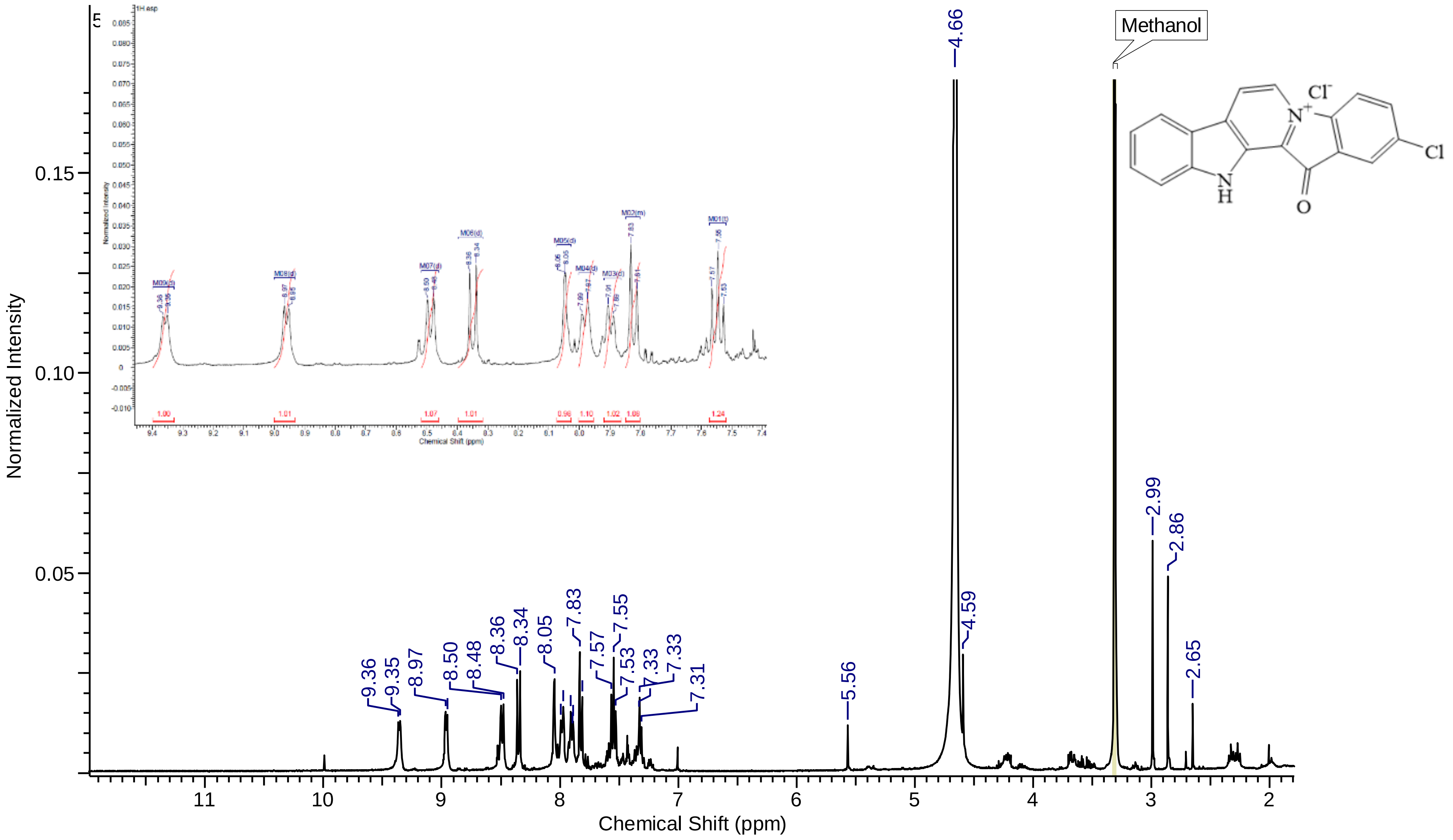

^{13}C NMR spectra of compound 21

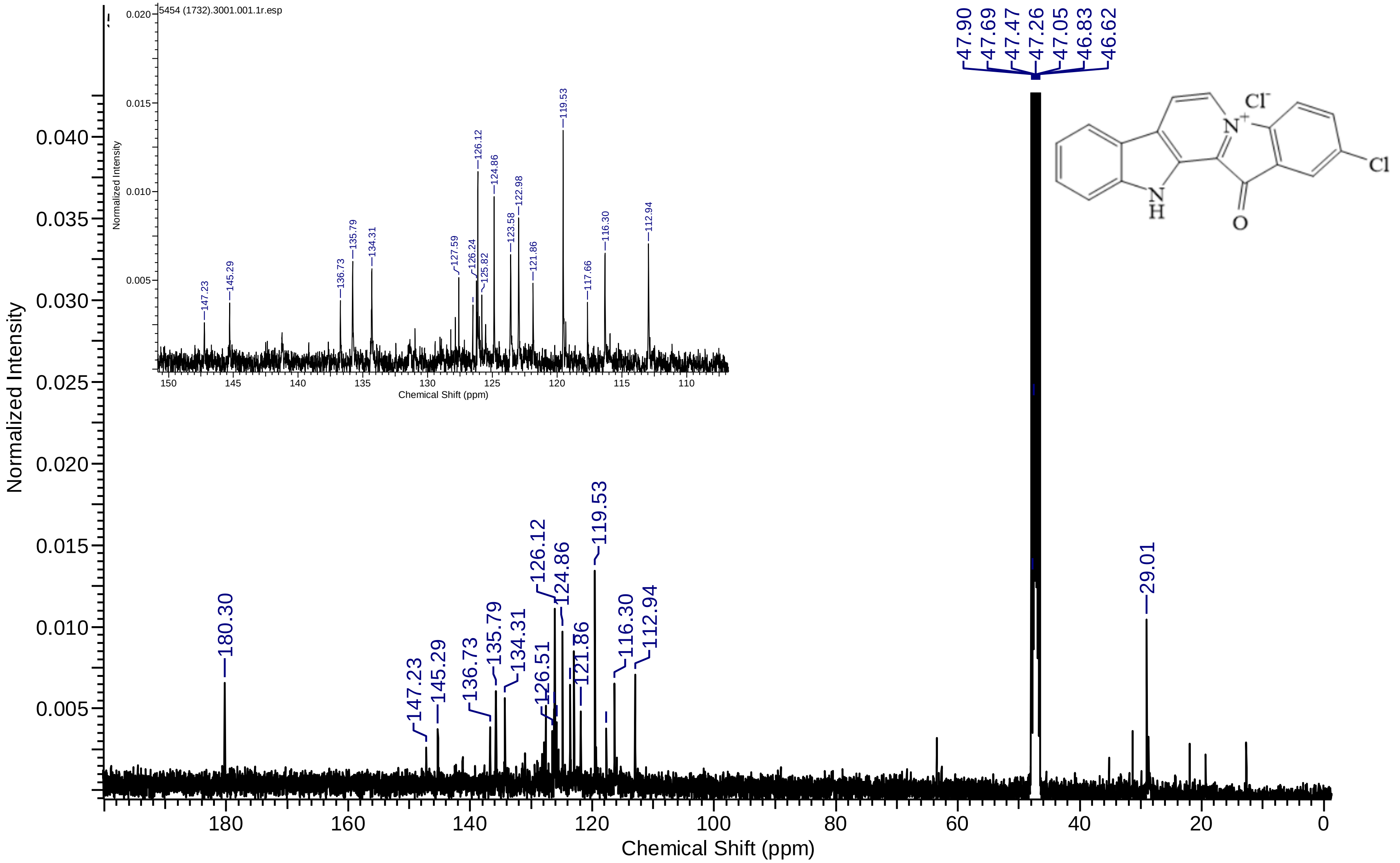

^{1}H NMR spectra of compound 10

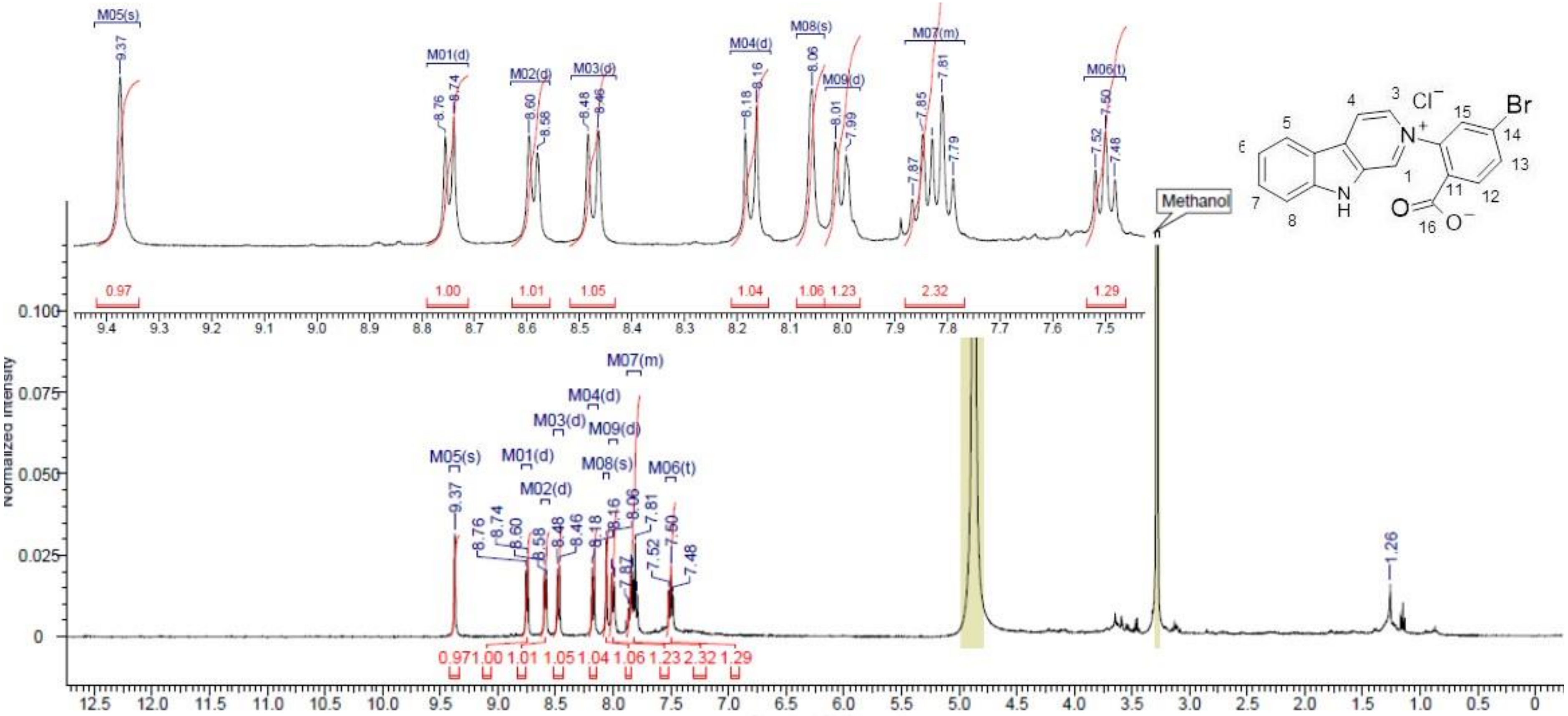

^{13}C NMR spectra of compound 10

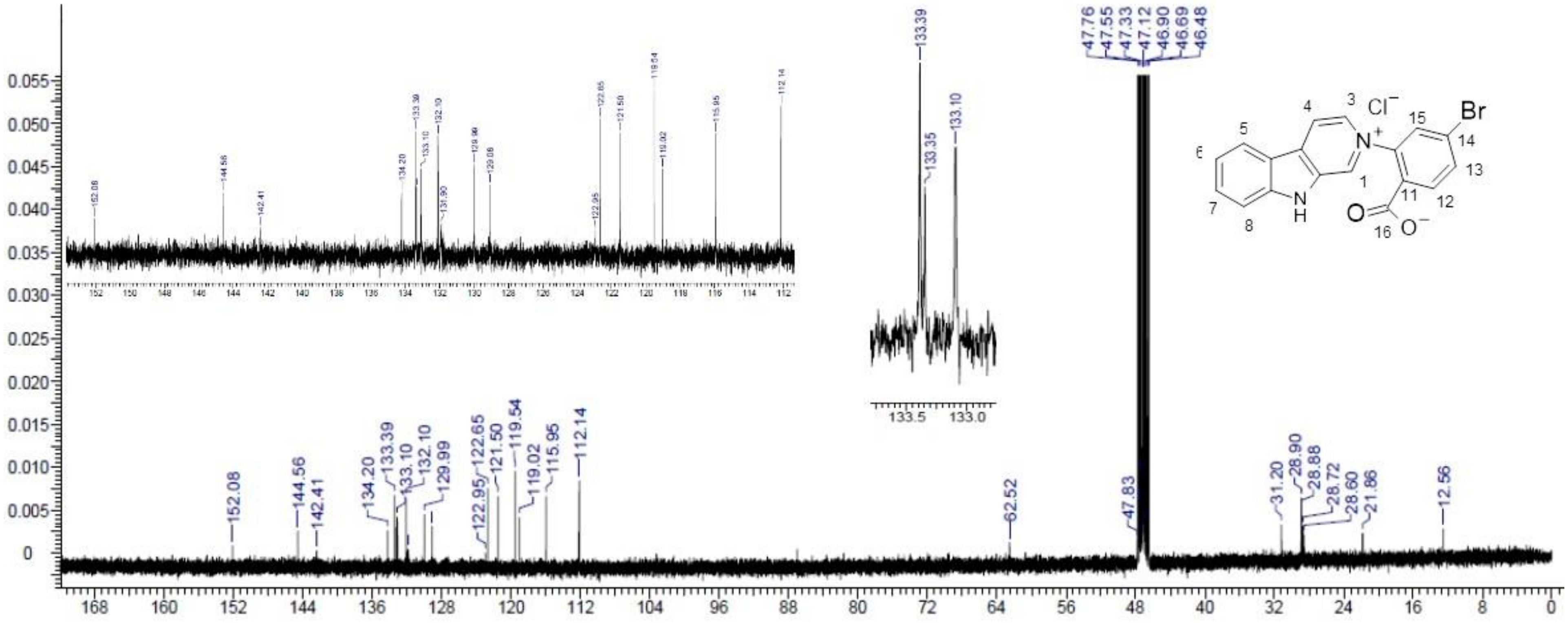

^{1}H NMR spectra of 14-bromoreticulatine (7)

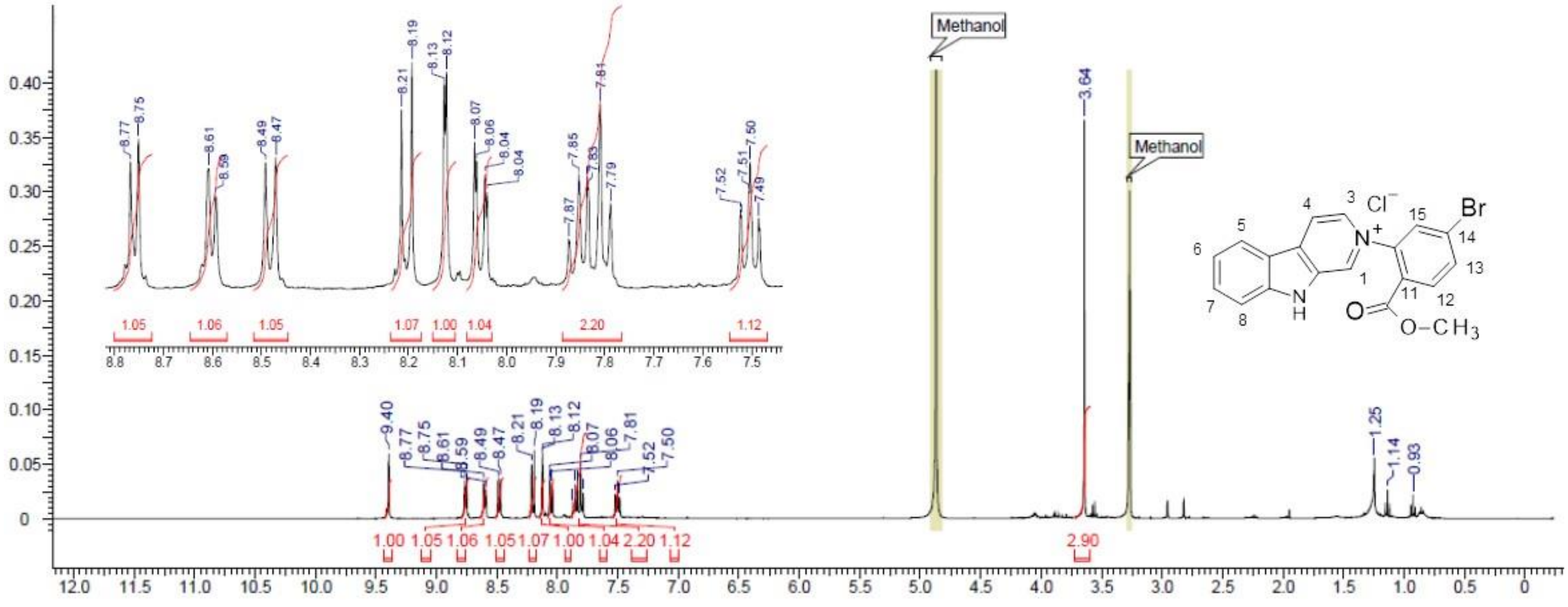

^{13}C NMR spectra of 14-bromoreticulatine (7)

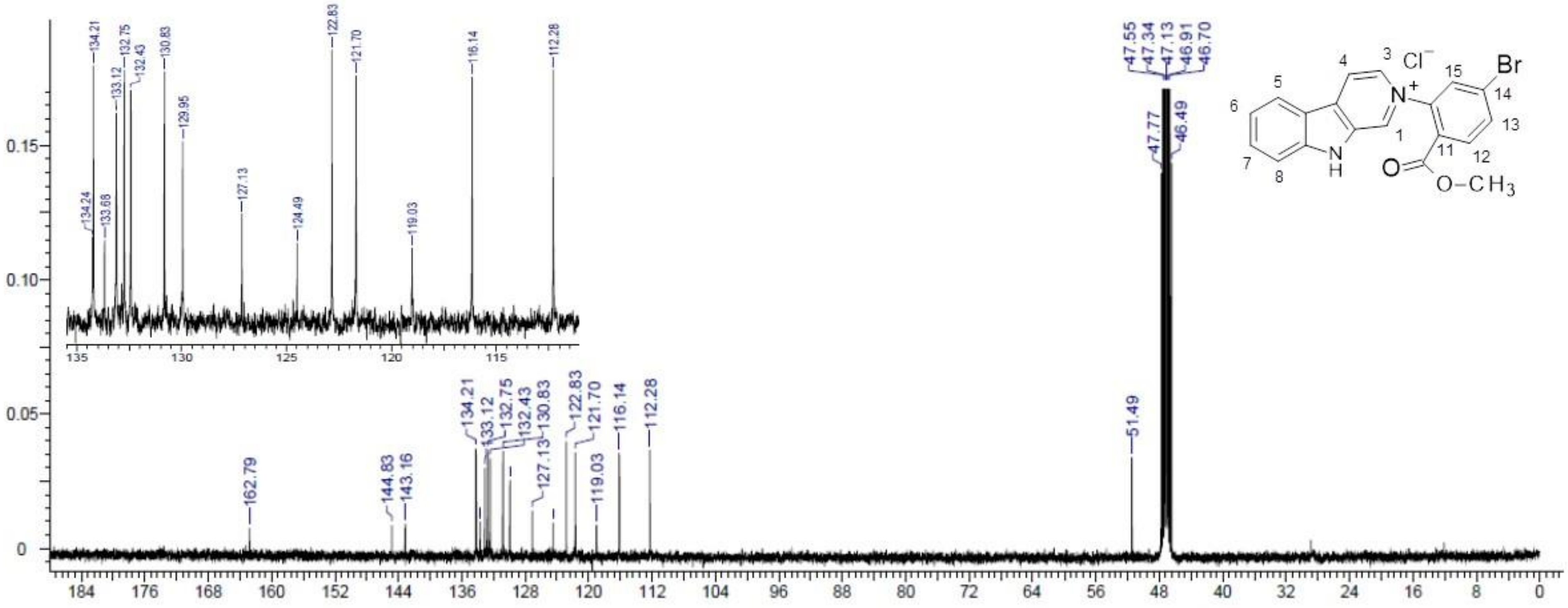

^{1}H NMR spectra of compound 23

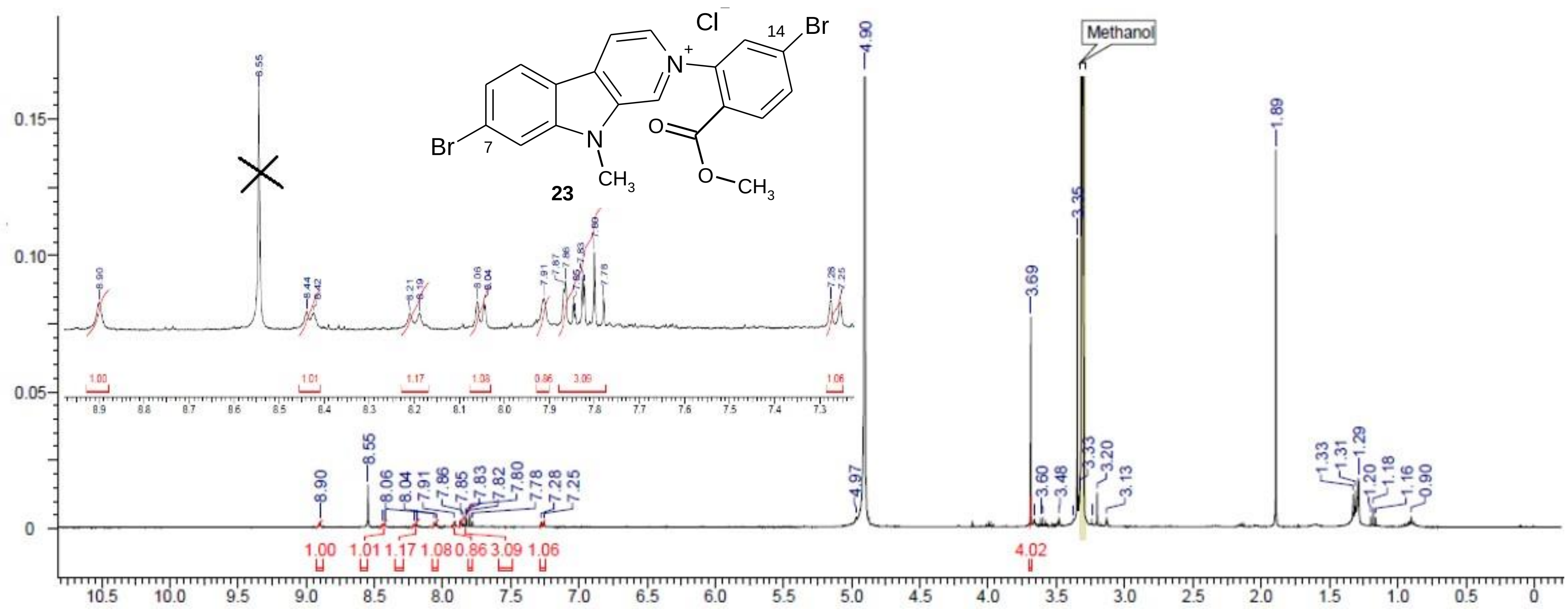

Supplement: Supplementary file 1 [file marinedrugs-17-00496-s001.pdf]
